# Exploration of the genomic atlas of Dof transcription factor family across genus *Oryza* provides novel insights on rice breeding in changing climate

**DOI:** 10.3389/fpls.2022.1004359

**Published:** 2022-11-03

**Authors:** Javaria Tabassum, Qasim Raza, Awais Riaz, Shakeel Ahmad, Muhammad Abdul Rehman Rashid, Muhammad Arshad Javed, Zulfiqar Ali, Fengyu Kang, Iqrar Ahmad Khan, Rana Muhammad Atif, Ju Luo

**Affiliations:** ^1^ State Key Laboratory of Rice Biology, China National Rice Research Institute, Hangzhou, China; ^2^ Department of Plant Breeding and Genetics, Faculty of Agricultural Sciences, University of the Punjab, Lahore, Pakistan; ^3^ Precision Agriculture and Analytics Lab, National Centre in Big Data and Cloud Computing, Centre for Advanced Studies in Agriculture and Food Security, University of Agriculture Faisalabad, Faisalabad, Pakistan; ^4^ Molecular Breeding Laboratory, Rice Research Institute, Kala Shah Kaku, Sheikhupura, Pakistan; ^5^ Department of Crop, Soil and Environmental Sciences, University of Arkansas, Fayetteville, AR, United States; ^6^ National Center for Genome Editing for Crop Improvement and Human Health, Centre for Advanced Studies in Agriculture and Food Security, University of Agriculture Faisalabad, Faisalabad, Pakistan; ^7^ Department of Bioinformatics and Biotechnology, Government College University Faisalabad, Faisalabad, Pakistan; ^8^ Department of Plant Breeding and Genetics, University of Agriculture Faisalabad, Faisalabad, Pakistan; ^9^ Institute of Horticultural Sciences, University of Agriculture, Faisalabad, Pakistan

**Keywords:** rice, DNA-binding with one finger, gene evolution, phylogenetics, comparative genomics

## Abstract

DNA-binding with one finger (Dof) transcription factors have been demonstrated to regulate various stresses and developmental processes in plants. Their identification and comparative evolutionary analyses in cultivated and wild species of genus oryza were yet to be explored. In this context, we report a comprehensive genomics atlas of DNA-binding with one finger (Dof) family genes in 13 diverse rice genomes (five cultivated and eight rice wild-relatives) through a genome-wide scanning approach. A galore of 238 *Dof* genes, identified across the genus *Oryza*, are categorized into seven distinct subgroups by comparative phylogenetic analysis with the model plant *Arabidopsis*. Conserved motifs and gene structure analyses unveiled the prevalence of species- and subgroups-specific structural and functional diversity that is expediating with the evolutionary period. Our results indicate that Dof genes might have undergone strong purifying selections and segmental duplications to expand their gene family members in corresponding *Oryza* genomes. We speculate that miR2927 potentially targets the Dof domain to regulate gene expression under different climatic conditions, which are supported by *in-silico* and wet-lab experiments-based expression profiles. In a nutshell, we report several superior haplotypes significantly associated with early flowering in a treasure trove of 3,010 sequenced rice accessions and have validated these haplotypes with two years of field evaluation-based flowering data of a representative subpanel. Finally, we have provided some insights on the resolution of *Oryza* species phylogeny discordance and divergence highlighting the mosaic evolutionary history of the genus *Oryza*. Overall, this study reports a complete genomic landscape of the Dof family in cultivated and wild *Oryza* species that could greatly facilitate in fast-track development of early maturing and climate-resilient rice cultivars through modern haplotype-led breeding.

## Introduction

Transcription factors (TFs) are regulatory proteins that bind to specific nucleotide sequences in the promoter region of target genes and regulate their expressions both at transcriptional and post-transcriptional levels (Riechmann et al., 2000). This regulation affects various downstream fundamental processes crucial for optimal growth and development. Therefore, identification as well as functional characterization of TFs are indispensable for understanding and exploiting their regulatory functions.

Among several known TF families, members of the DNA-binding with one finger (Dof) subfamily are plant-specific proteins belonging to the zinc finger multigene superfamily (Noguero et al., 2013). Dof proteins are distributed throughout the plant kingdom; from simple unicellular to complex multicellular organisms (Moreno-Risueno et al., 2007). N-terminal of these proteins contains a single copy of 52 amino acids long highly conserved core Dof domain, whereas C-terminal contains a less conserved transcriptional activation domain. The Dof domain forms a C_2_-C_2_ zinc finger motif that binds to the AAAG cis-regulatory element in the promoter region of target genes (Yanagisawa, 2016). Earlier evidence suggests that besides DNA binding activity, Dof proteins also participate in protein-protein interactions and physically interact with other transcription factor families such as bZIP, MYB, WRKY, and ZFP (Yanagisawa, 2002; Isabel-LaMoneda et al., 2003; Zou et al., 2008; Wei et al., 2016). Dof TFs play multifarious roles in plants including cell signaling (Riechmann et al., 2000), hormonal signaling (Moreno-Risueno et al., 2007), differentiation of guard cells (Negi et al., 2013), fatty acid metabolism (Ibáñez-Salazar et al., 2014), flower abscission (Wei et al., 2010), formation of interfascicular cambium (Guo et al., 2009), photoperiodic flowering (Fornara et al., 2009), nitrogen assimilation (Wang et al., 2013), regulation of flowering time (Liu et al., 2020), seed and seed development (Ruta et al., 2020), and tolerance against abiotic and biotic stresses (Bhullar et al., 2007; Liu et al., 2019). Keeping in view their diversified roles in biological and physiological processes, it is imperative to explore these proteins in the genomes of important crop species and gain insights into their evolutionary patterns.

Rice (*Oryza sativa* L.) is one of the oldest and most consumed cereal crops. More than 50% of the world’s population solely depends upon it to fulfill their daily calorie requirements (Fukagawa and Ziska, 2019; Raza et al., 2020). The *Oryza* genus contains at least 22 diploids (2n = 2X = 24) and 5 tetraploids (2n = 4X = 48) species, which originated from a common wild grass ancestor about 130 million years ago and spread to different parts of the world (Sangeetha et al., 2020). Among all known rice species, only two diploid species *Oryza sativa* (Asian rice) and *Oryza glaberrima* (African rice) are cultivated around the world. *O. sativa* is cultivated on more than 90% of the total world area under rice cultivation and the majority of it is produced and consumed in Asian countries (Fukagawa and Ziska, 2019). Rice also serves as a model cereal crop due to its small genome size, ease of genetic transformation, and genomic synteny with other important grass species (Jackson, 2016). The draft genome sequences of two subspecies of *O. sativa* (*indica* and *japonica*) were completed in 2002 (Goff et al., 2002; Yu et al., 2002) and high-quality genome annotation of *japonica* rice was available in 2004 (Matsumoto et al., 2005), which was later improved in 2013 (Kawahara et al., 2013) and serves as a foundation of cereal genomics (Matsumoto et al., 2016). The genomes of *O. glaberrima* (Wang et al., 2014) and several other cultivated and wild rice species have also been sequenced (Stein et al., 2018). Comparative genomic studies among sequenced rice genomes would help investigations on evolution and genomic diversity among *Oryza* species and could provide deeper insights into crop improvement under rapidly changing global environments (Raza et al., 2020).

Genome-wide analysis of Dof family TFs has been reported in several plant species such as *Arabidopsis* and rice (Lijavetzky et al., 2003), sorghum (Kushwaha et al., 2011), cotton (Chattha et al., 2020) and olive (Mariyam et al., 2021). However, a comparative genome-wide analysis of Dof family TFs among cultivated and wild rice relatives is missing so far. With the advent of next-generation sequencing technologies, many cultivated rice cultivars and wild relatives have been sequenced in the recent past (Stein et al., 2018; Wang et al., 2018). The availability of this genomic data provides an opportunity to perform comparative genomic studies and gain new insights into the genomic diversity and evolutionary patterns of *Oryza* species. So far, only a single comparative study of Dof family transcription factors has been reported in cultivated and ancestral cotton species (Chattha et al., 2020). Here, in this study, we performed comprehensive whole-genome analyses and identified Dof family TFs from 10 cultivated and wild rice species including *O. sativa* (*indica* and *japonica*), *O. nivara*, *O. rufipogon*, *O. glaberrima*, *O. barthii*, *O. glumipatula*, *O. meridionalis*, *O. punctata*, and *O. brachyantha*. Based upon comparative phylogenetic analyses, the rice Dof TFs were classified into different subgroups. Gene structures, conserved motifs, and gene duplication analyses were also performed to understand the evolutionary patterns. Furthermore, promoter analysis, prediction of miRNA target sites and *in-silico*, as well as wet-lab experiments-based expression analyses in different tissues and conditions, were also investigated. Moreover, superior haplotypes for earliness breeding were also identified along with resolving the complex evolutionary phylogenetic relationships among closely related species. In conclusion, this study provides a complete genomic landscape of Dof family TFs in ten *Oryza* genomes that could be further exploited for wider cultivation and crop improvement by humans.

## Material and methods

### Database search, sequence retrieval, and identification of *Dof* genes

The complete genome assemblies along with annotation files of four *Oryza sativa indica* genotypes i.e. 93-11, Minghui 63 (MH63), Zhenshan 97 (ZS97), and Shuhui498 (R498) were retrieved (**Supplementary Table S1**) for the identification of Dof encoding genes across these genomes. Moreover, genome assemblies of nine other *Oryza* species including *O. sativa japonica*, *O. barthii*, *O. brachyantha, O. glaberrima*, *O. glumipatula*, *O. meridionalis*, *O. nivara*, *O. punctata*, and *O. rufipogon* were were also downloaded (**Supplementary Table S1**) for further comparative analysis of *Dof* genes. These downloaded genome sequences were thoroughly examined for the identification of Dof family members in all given rice species using CLC Sequence Viewer version 7 (CLC Bio, QIAGEN A/S, Aarhus, Denmark). The presence of the conserved Dof domain was further validated through a conserved domain search tool (accessed online at https://www.ncbi.nlm.nih.gov/Structure/cdd/cdd.shtml) (Marchler-Bauer et al., 2015). The molecular weight and isoelectric points of all the identified *Dof* genes were computed using Expasy Compute pI/Mw web tool (https://web.expasy.org/compute_pi/) (Gasteiger et al., 2003). The renaming of identified genes was done according to the *Oryza* species-specific chromosomal location information as adapted for other plant species (Yanagisawa, 2002; Chattha et al., 2020 **Supplementary Table S3**). For instance, *OsInDof1.5* refers to the fifth *Dof* gene present on chr. 1 of *O. sativa indica*. (**Supplementary Table S4**).

### Identifying variation of Dofs among four *O. sativa indica* genomes

The protein sequences of all the identified Dofs from four genomes of indica rice were aligned using Clustal Omega (Sievers et al., 2014; accessed at https://www.ebi.ac.uk/Tools/msa/clustalo/) using default parameters. The resultant multiple sequence alignment was used for constructing a phylogenetic tree using the neighbor-joining method employing molecular evolutionary genetics analysis software (MEGA-X) (Kumar et al., 2018). The similarities and differences among the *Dofs* from each of the four genomes were visualized using a Venn diagram through a webtool (accessed at https://bioinformatics.psb.ugent.be/webtools/Venn/).

### Comparative phylogenetic analysis of *Dofs* from ten *Oryza* species and identification of paralogous and orthologous gene pairs

The Dof protein sequences of *Arabidopsis* (Yanagisawa, 2002) and ten *Oryza* species were subjected to multiple sequence alignment using MAFFT (v7) (accessible at https://mafft.cbrc.jp/alignment/server/) (Katoh et al., 2018) by choosing L-INS-i algorithm with default parameters. The maximum likelihood (ML) comparative phylogenetic tree was inferred with IQ_Tree_ (Nguyen et al., 2015) by choosing JTTDCMut+F+I+G4 best fit substitution model (Kalyaanamoorthy et al., 2017) according to the Bayesian information criterion. The consistency of the ML tree was validated by setting an Ultrafast bootstrap value of 1000 (Hoang et al., 2018). The final phylogenetic tree was visualized with MEGA-X (Kumar et al., 2016). The Dof genes were classified into different groups by adopting the classification scheme of Yanagisawa (2002). Paralogous and orthologous genes were assigned based on the results of comparative phylogenetic analysis.

### Gene structure and conserved motif analysis

The GFF3 files of all *Oryza* species were subjected to TBtools for displaying the gene structures (Chen et al., 2020). For conserved motifs analysis, peptide sequences of all *Oryza* species *Dof* genes were uploaded in MEME v5.4.1 (Bailey et al., 2009) with the following parameters: motif distribution, zero or one occurrence per sequence; the number of motifs, 10; motifs width, 6–200, while all other parameters were kept default. The identified motifs were mapped onto the peptide lengths of all *Dof* genes with TBtools (Chen et al., 2020). The detailed information of identified motifs in all *Oryza* species is given in **Supplementary Table S5**.

### Gene duplication and divergence analysis

Coding sequences of all identified Dof genes were blasted against each other using Sequence Demarcation Tool V1.2 (Muhire et al., 2014) for computing the sequence similarities. Gene pairs with approximate≥ 90% similarity (E value <1e^-10^) were considered as duplicated genes and retained for further analysis. Furthermore, the CDS sequences of duplicated gene pairs were aligned with ClustalW in MEGA7 and subjected to Synonymous Non-synonymous Analysis Program V2.1.1 (www.hiv.lanl.gov/content/sequence/SNAP/SNAP.html) to compute the substitution rates. The ratio of non-synonymous to synonymous substitutions was also calculated to assume which type of codon selection operated on duplicated gene pairs during their evolution. Moreover, the approximate divergence time between duplicated gene pairs was also computed by using the formulae T=Ks/2r × 10^-6^ assuming a substitution rate (r) of 6.5 × 10^-9^ substitutions/synonymous site/year (El Baidouri et al., 2017). Finally, a circular diagram showing the links between duplicated gene pairs among ten *Oryza* genomes was created with shiny Circos (Yu et al., 2018). The detailed information of all duplicated gene pairs is given in **Supplementary Table S6**.

### Identification of cis-regulatory elements through promoter analysis

The 2 kb upstream sequences from the transcription start site of all Dof genes were retrieved from the Regulatory Sequence Analysis Tool homepage (http://rsat.eead.csic.es/plants/index.php) (Nguyen et al., 2018) by searching through corresponding gene IDs. To prevent overlap with neighboring genes, the sequences were clipped when a predicted gene is located within the promoter region. Cis-acting regulatory elements (CAREs) were identified by uploading the promoter sequences in PlantCARE database with default parameters (Lescot et al., 2002). The CAREs without any putative function/role were discarded, as well as those involved in core transcription initiation function in eukaryotes such as TATA and CAAT/CAT boxes. The identified CAREs were mapped onto the promoter regions of all *Oryza* species Dof genes with TBtools (Chen et al., 2020). The detailed results of promoter analysis are given in **Supplementary Table S7** and summarized in **Supplementary Table S8**.

### miRNA target site prediction

miRNAs potentially targeting the identified Dof genes in two cultivated rice genomes (*O. sativa indica* and *japonica*) were predicted by uploading their transcript sequences on the web-based psRNA Target Server (https://www.zhaolab.org/psRNATarget/) (Dai et al., 2018). Stringent selection criteria with a penalty score of ≤2.0 were adopted for the identification of targets using the already reported rice miRNA database available at the above-mentioned web server. Only frequently occurring miRNAs were considered for creating the final miRNA target site prediction figure.

### *In-silico* expression analysis

The normalized (Log_2_ fold change) expression profiles in root and shoot tissues of *O. sativa japonica* in response to six plant hormones including abscisic acid, auxin, brassinosteroid, cytokinin gibberellin, and jasmonic acid were retrieved from the RiceXPro database (http://ricexpro.dna.afrc.go.jp) (Sato et al., 2013). These data were collected from 7-days old rice seedlings individually treated with different plant hormones and incubated for different periods. Details of expression analysis and normalization were described by Sato et al. (2013). Similarly, *O. sativa indica* expression data of low light-sensitive (IR8) and tolerant (Swarnaprabha) rice genotypes were mined from Sekhar et al. (2019). Briefly, both rice genotypes were exposed to low light treatments for one day (T1), three days (T3), and five days (T5) along with control treatments during the active tillering stage (40 days after germination) before the final expression analysis. Finally, the heatmaps of retrieved expression data were generated with TBtools (Chen et al., 2020).

### Plant material and stress treatments

An *indica* rice cultivar “9311” with sequenced genome data was used for gene expression studies under high temperature, low temperature, and low light stress conditions. Seedlings were grown for three weeks under controlled laboratory conditions at 27 ± 2°C with a relative humidity of 60% under a photoperiod of 14h/10h (day/night) before the application of stress treatments. High temperature (40 ± 1°C), low temperature (15 ± 1°C), and low light intensity (20% of control; 60 µmolm^-2^s^-1^) were administered for one week during 14h daylight periods only. Leaf and stem tissues were harvested from control and treated seedlings and stored at -80°C for subsequent RNA isolation.

### RNA isolation and quantitative real-time PCR

Total RNA was isolated and reverse transcribed using RNA-prep pure plant kit (TIANGEN) and HiScript II QRT SuperMix (Vazyme), respectively (Chun et al., 2020). A quantitative real-time polymerase chain reaction (qRT-PCR) for 14 *OsInDof*s was performed using SYBR green mixture on an ABI 7500 RT-PCR detection system. Rice *Ubiquitin* was used as internal control and for normalization of expression data as described previously (Chun et al., 2020). The forward and reverse primer sequences of all genes are listed in **Supplementary Table S10**.

### Haplotype analysis

The 3K rice genomes (RG) variation data comprising 3,010 rice accessions belonging to indica, japonica, aus/boro, aromatic basmati/sadri, and admixture groups was mined through the rice functional genomics & breeding (RFGB v2.0) database (Sun et al., 2017; Wang et al., 2020). In the 3K RG panel, several haplotypes have been predicted using single nucleotide polymorphisms (SNPs) based on non-synonymous substitutions in the coding region of protein-encoding genes. By choosing *O. sativa japonica* Dof genes having orthologs in all other studied *Oryza* genomes, we selected only those haplotypes which were significantly associated with days to heading phenotypic trait (Wang et al., 2020) (**Supplementary Table S11**).

### Field evaluation of the 3K RG representative sub-panel

A sub-panel of 22 rice accessions belonging to all major and minor groups of the 3K RG panel was selected for field evaluation (**Supplementary Table S12**). Thirty-days old seedlings of sub-panel accessions were transplanted in the puddled field at a distance of 22.5 × 22.5 cm with a fertilizer dose of 116:87:62 (NPK) during cropping seasons 2020 and 2021 at the experimental area of Rice Research Institute, Kala Shah Kaku, Pakistan. Standard agronomic and plant protection practices were followed during both growing seasons. Two rows of each accession (32 plants) were considered as two independent replications and days to heading data (from sowing date) were recorded from five randomly selected guarded plants from each row. Averaged data of both replications and seasons were considered for haplotype validation.

## Results

### Identification and comparison of *Dof* genes in four important *indica* Rice genomes

**Figure 1:**
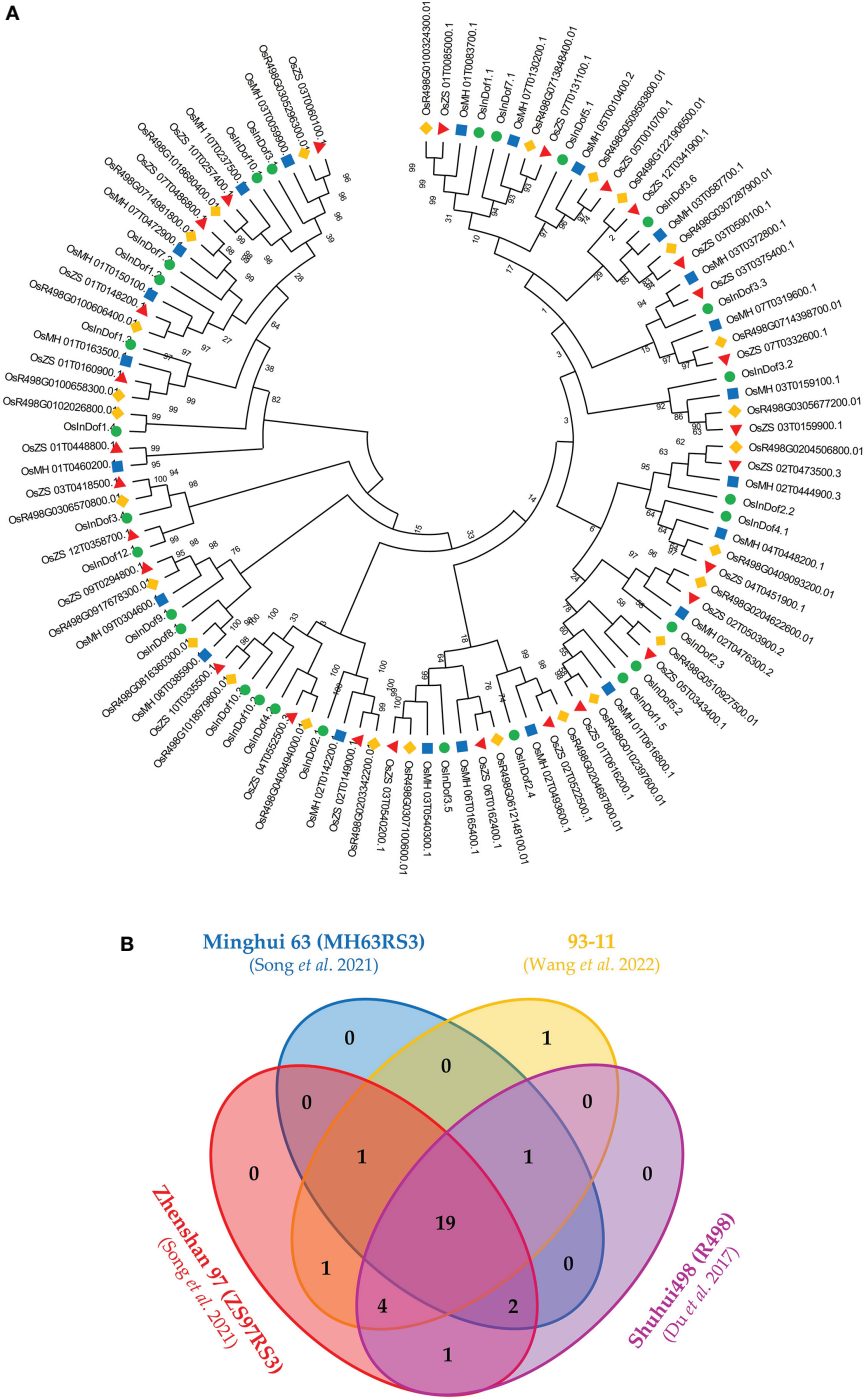
Comparison of Dof proteins among four elite genotypes (93-11, MH63, ZS97, and R498) of *O. sativa indica*. **(A)** The comparative phylogenetic tree of Dofs from four elite genotypes of indica rice. The evolutionary history was inferred using the Neighbor-Joining method (Saitou and Nei, 1987). The evolutionary distances were computed using the Poisson correction method (Zuckerkandl and Pauling, 1965) and are in the units of the number of amino acid substitutions per site. This analysis involved 105 protein sequences. All positions containing gaps and missing data were eliminated (complete deletion option). There was a total of 51 positions in the final dataset. Evolutionary analyses were conducted in MEGA X (Kumar et al., 2018). **(B)** Comparison of presence of Dof protein among four indica rice elite genotypes through Venn diagram.

To gain insight into the evolution and expansion of Dof TF-encoding genes in *O. sativa indica*, the genomes of four important *indica* varieties (93-11, MH63, ZS97, and R498) were thoroughly scanned. The systematic analysis revealed that ZS97 genome harbored the highest number of non-redundant *Dof* genes i.e 28 genes. On the contrary, only 23 *Dof* genes were identified MH63 genome. Both 93-11 and R498 genomes contained 27 Dof genes. Further comparative analysis revealed the presence of orthologs of 19 common Dof genes in all the four *indica* genomes (**Figure 1A**; **Supplementary Table S2**). The ZS97 genome does not contain any Dof gene on chromosome no. 8 while remaining all three genomes showed the presence of a single locus on chr. 8 (**Figure 1B**). Contrarily, the 93-11 genome did not contain any gene on chr. 6 as compared to the other three genomes. An extra Dof gene on chr. 10 of 93-11 genome seems to have resulted from segmental duplication. Overall, the gene duplication might have played role in the expansion of *Dof* family members in ZS97 and 93-11 genomes. Copy number variation of some *Dof* genes on chr. 5, 7 10 and 12 have been observed in these indica rice genomes. For further comparative analysis, the *Dof* genes identified from 93-11 genome were used as representative of *O. sativa indica* group.

### Identification, classification, and distribution of Dof genes in ten *Oryza* species

Through comprehensive genome-wide scanning of whole genomes by using the Dof domain amino acids sequence as a query, a total of 250 Dof domain encoding genes were identified in 10 rice genomes. After excluding incomplete Dof domain-containing and non-annotated genes, 238 non-redundant and full-length protein encoding Dof genes were considered for further analyses (**Supplementary Table S3**).

**Figure 2:**
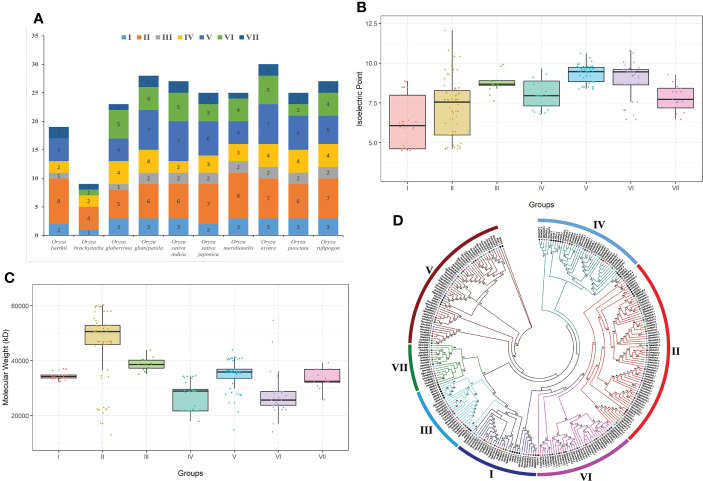
Classification and distribution of Dof genes among ten *Oryza* genomes. **(A)** Distribution of Dof genes in ten *Oryza* genomes. Each bar represents an individual genome with values on top of bars indicating the total number of Dof genes in that genome. **(B)** Distribution of iso-electric points and **(C)** molecular weights of Dof proteins belonging to different subgroups, respectively. **(D)** Comparative maximum likelihood phylogenetic tree of *Arabidopsis* and *Oryza* species Dof genes inferred with IQ_Tree_ software (Nguyen et al., 2015) after MAFFT alignment (Katoh et al., 2018) of amino acid sequences. Dof subgroups are indicated with different colors. *Arabidopsis* Dof genes are labeled with solid black circles, whereas *Oryza* species Dof genes are labeled with species-specific triangles of different colors. Bootstrap values (≥50%) as calculated from 1,000 replicates are displayed at tree nodes.

The *O. nivara* genome assembly contained the highest (30) number of Dof genes, followed by *O. glumipatula* (28), *O. sativa indica* (27), *O. rufipogon* (27), *O. sativa japonica* (25), *O. meridionalis* (25), *O. punctata* (25), *O. glaberrima* (23) and *O. barthii* (19) (**Figure 2A**). Whereas the *O. brachyantha* genome harbored the least number (9) of Dof genes. Furthermore, the *Dof* groups II, V, and VI members were overrepresented, whereas groups III and VII members were less represented in all these genomes. Interestingly, *O. brachyantha* and *O. barthii* genomes lack Dof members of at least two (III and V) and one (VI) subgroups, respectively (**Figure 2A**), which could have been lost during the speciation events in rice crop evolution.

A comparative maximum likelihood (ML) phylogenetic tree was inferred with IQ_Tree_ using *Arabidopsis* and 10 rice genomes *Dof* genes to explore the evolutionary relationships among identified genes (**Figure 2D**). The classification of *Dof* genes in the resultant phylogenetic tree was carried out folowing classification scheme proposed by Yanagisawa (2002). All rice Dof genes were classified into seven different groups. Group II contained the highest number of genes (64; ~27%), followed by group V (50; ~21%), group VI (33; ~14%), group IV (32; ~13%), and the group I (26; ~11%), whereas groups VII (17; ~7%) and III (16; ~7%) harbored the least number of Dof genes. In general, the genes of 10 rice genomes showed more sequence homology among them as compared with *Arabidopsis*, as *Arabidopsis* Dof genes were clustered into separate clades showing distant orthologous relationships with rice genes (**Figure 2D**). This classification of rice Dof genes into seven different groups might be due to group-specific conserved amino acid motifs among the protein sequences of specific group members. Collectively, these results suggest that different Dof genes might function within different conditions, which is supported by genetic differences in their isoelectric points (**Figure 2B**) and molecular weights (**Figure 2C**).

### Conserved motifs and gene structures of different Dof subgroups

**Figure 3:**
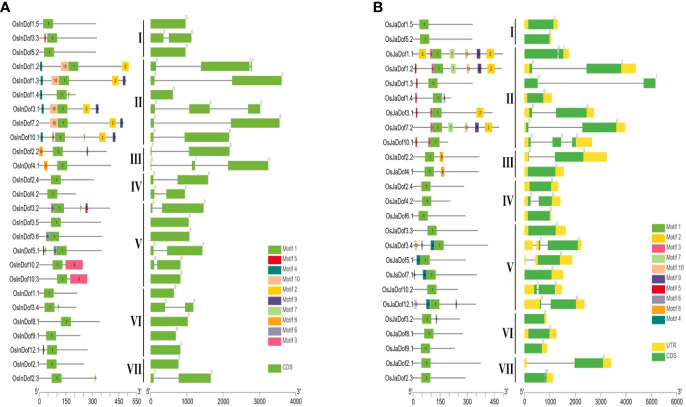
Conserved motifs and gene structures of Dof genes in **(A)** *indica* and **(B)** *japonica* rice. Conserved motifs were identified by uploading amino acid sequences to the MEME online tool (Bailey et al., 2009) and displayed on protein lengths with TBtools (Chen et al., 2020). Similarly, gene structures were drawn on genomic lengths by using respective GFF3 files of both rice types. Sub-groups are indicated with Roman numbers (I−VII) in the middle of both figures.

The amino acid sequences of all Dof genes were explored to predict conserved motifs among them. All identified genes contained at least one Dof domain in their protein sequences (**Figure 3** and **Supplementary Figures S1A–S1H**, **Supplementary Table S5**). Members of group II contained the highest number of motifs (2–10), whereas members of groups I and VII contained the least number of motifs (1–3). All other groups contained a variable number of motifs (1–8). In general, members of the same subgroups shared conserved motifs, whereas substantial differences were observed among members of different groups supporting the evolutionary phylogenetic relationships.

Similarly, gene structures were drawn to explore structural differences among different Dof subgroups and genes. A highly significant number of genes (~92%) are either intronless or one intron, with only < 6% and < 2% genes containing two and three introns, respectively (**Figure 3**; **Supplementary Figures S1A–S1H**; **Supplementary Table S3**). Whereas, only two genes harbored four introns in their structures. Collectively, these results suggest potential structural and functional conservation among members of the same subgroups but diversification between members of different subgroups.

### Gene duplications and evolution of Dof genes

**Figure 4:**
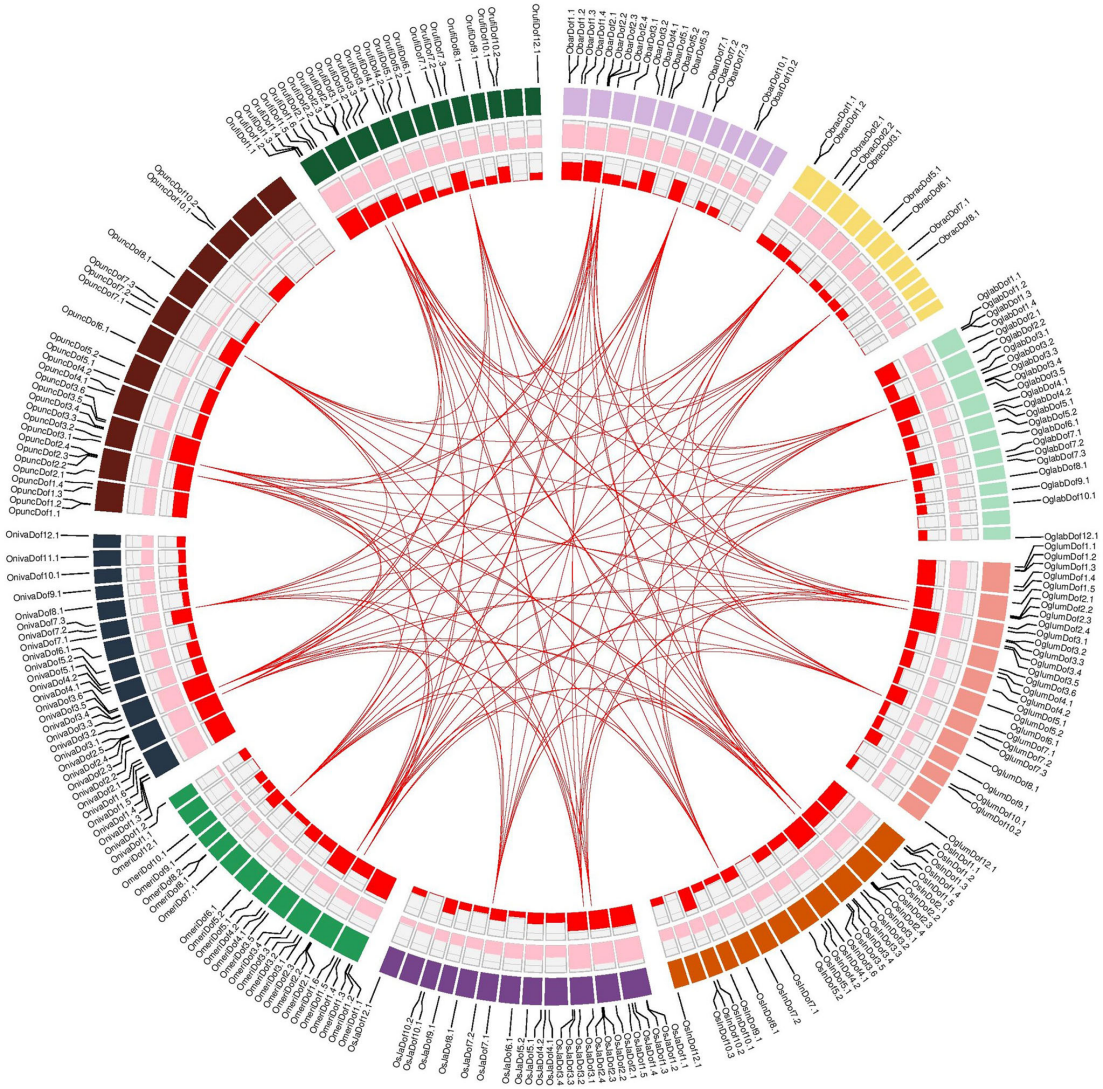
Genomic landscape of Dof genes among ten *Oryza* genomes. Circular diagram from outside to inside are gene names and locations on individual species-specific colored chromosomal bands, density of high confidence protein-encoding genes (count/Mb; min = 65, max = 133), density of Dof genes (count/Mb; min = 0.0, max = 0.17) and links indicating duplicated genes among ten rice species. Detailed information about duplicated gene pairs is provided in **Table S6**.

Duplicated Dof gene pairs were identified by comparing the coding sequences and those pairs showing ≥90% sequence similarity were considered as duplicated. A total of 136 duplicated gene pairs corresponded to 30 unique Dof genes of ten *Oryza* genomes (**Figure 4**; **Supplementary Table S6**). Interestingly, all these duplicated gene pairs were sub-group specific, only identified in subgroups II and VII, and exclusively located on chromosomes 2, 3, and 7. We also identified at least three duplicated gene pairs among closely related *Oryza* genomes with 100% identical coding sequences. The duplicated gene pairs among all *Oryza* genomes ranged from 2 to 5 with the highest duplicated pairs found between *O. barthii* and *O. meridionalis* (**Supplementary Table S6**).

Furthermore, we also computed substitution rates among duplicated gene pairs to investigate different evolutionary patterns. In general, Dof genes were under strong purifying selections as nearly 85% of the duplicated pairs had a Ka/Ks ratio < 1, whereas only 12% of pairs were under positive selections (Ka/Ks > 1) (**Supplementary Table S6**). Moreover, all these duplicated genes were estimated to be diverged between 0.23 to 24.50 million years ago (MYA). Collectively, these results suggest that purifying selections are operating on codons to expand the Dof family members in *Oryza* genomes.

### Cis-acting regulatory elements in promoter regions of Dof genes

Cis-acting regulatory elements (CAREs) are found in the promoter region of a gene which binds to the transcription factors and regulates the gene expression. A variety of CAREs were predicted in the promoters of Dof genes of all *Oryza* genomes using the PlantCARE database (**Supplementary Table S7**). All these CAREs corresponded to diverse biological functions including abiotic and biotic stresses, plant hormones, and growth and development-related processes. Interestingly, the promoters of all identified Dof genes were extremely enriched with light, abscisic acid, methyl jasmonate, and anaerobic induction responsive CAREs (**Supplementary Figures S2A–S2J**; **Supplementary Table S8**). These results indicate that Dof family members can regulate diverse biological and physiological processes with highly significant regulatory roles in light responsiveness and hormone signaling.

### miRNAs targeting the *O. sativa Dof*s

**Figure 5:**
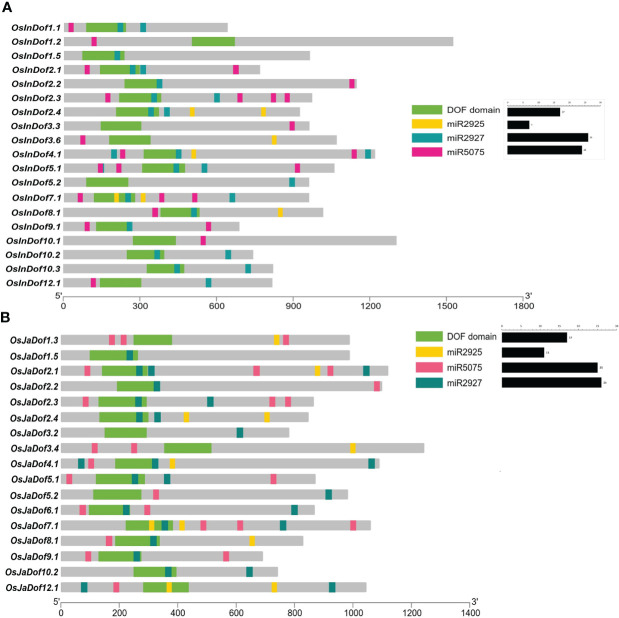
miRNA’s potentially targeting **(A)** *indica* and **(B)** *japonica* Dof genes. Previously reported information related to rice miRNAs and their potential targets was retrieved from the psRNA Target Server (Dai et al., 2018) and target sites were mapped onto the transcripts of Dof genes with TBtools (Chen et al., 2020).

Various miRNAs post-transcriptionally target several transcription factor families in different crops to regulate the gene expression patterns. Here, we identified several kinds of miRNAs which can potentially target the transcripts of identified Dof genes of the two most common *Oryza sativa* subspecies (*indica* and *japonica*) (**Supplementary Table S9**). Among identified miRNAs, three types (osa-miR2925, osa-miR2927 and osa-miR5075) were most frequent as the transcripts of 19 *indica* (~70%) and 17 *japonica* (68%) Dof genes contained the putative binding sites of these miRNAs (**Figure 5**). Surprisingly, osa-miR2927 was observed to be potentially targeting the Dof domain sequence of nearly 48% of both *O. sativa* sub-species genes. Likewise, osa-miR2925 potentially targets the Dof domain in a few gene transcripts and predominately the downstream regions of the Dof domain in all genes, whereas osa-miR5057 only targets the upstream or downstream regions of the Dof domain in all genes of both sub-species (**Figure 5**). Collectively, these results highlight that *Dof* genes are frequent targets of several miRNAs with significant roles of osa-miR2927, osa-miR2925, and osa-miR5075 in post-transcriptional regulation of Dof genes expressions.

### Expression profiles of *Dof*s under phytohormones, low light and temperature stress conditions

**Figure 6:**
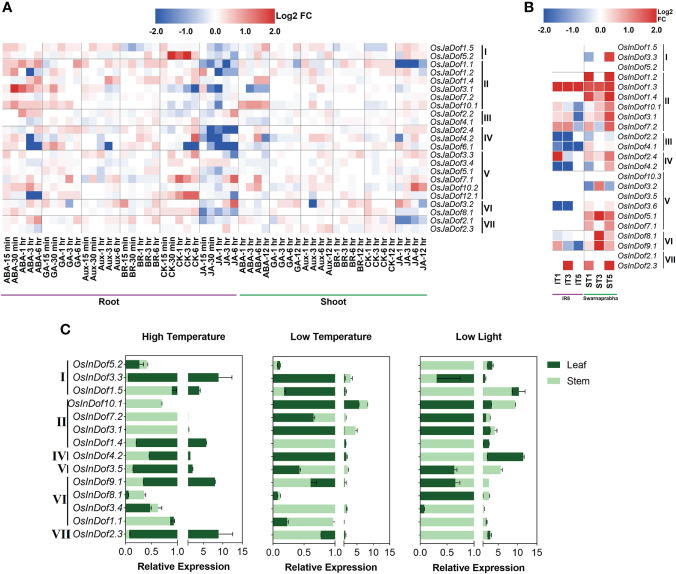
Expression profiles of Dof genes under stressful conditions. **(A)** RNA-seq-based normalized (Log_2_ fold change) expression patterns of *O. sativa japonica* Dof genes in phytohormones treated roots and shoots. Hormone treatments are listed in columns and Dof genes in rows. Roman numbers on the left of the gene names indicate different Dof sub-groups. Abbreviations; ABA (abscisic acid), GA (gibberellic acid), Aux (auxin), BR (brassinosteroid), CK (cytokinin), JA (jasmonic acid). **(B)** RNA-seq-based normalized (Log_2_ fold change) expression patterns of *O. sativa indica* Dof genes in response to low light treatments in susceptible (IR8) and tolerant (Swarnaprabha) rice genotypes. Abbreviations: IT1 (IR8 treated with low light for one day), IT3 (IR8 treated with low light for three days), IT5 (IR8 treated with low light for five days), ST1 (Swarnaprabha treated with low light for one day), ST3 (Swarnaprabha treated with low light for three days) and ST5 (Swarnaprabha treated with low light for five days). **(C)** Relative expression profiles of *O. sativa indica* Dof genes after one week of high temperature (40 ± 1°C), low temperature (15 ± 1°C), and low light (20% of control; 60 µmolm^-2^s^-1^) treatments during 14h daylight periods in 93-11 *indica* rice seedlings.

Comprehensive expression profiling could provide clues toward the understanding of gene functions. Here, we examined the global gene expression patterns of *O. sativa japonica* Dof genes in root and shoot tissues in response to six plant hormones (**Figure 6A**). In general, the majority of the genes (~68%) were deregulated after plant hormone treatments. As expected, nearly all *OsJaDof*s were highly deregulated in roots and shoots of jasmonic acid and abscisic acid-treated seedlings. Interestingly, these genes were significantly downregulated in response to jasmonic acid but up-regulated in response to abscisic acid treatments in root tissues (**Figure 6A**), however, unevenly deregulated in shoot tissues. Similarly, all these genes are inconsistently and marginally deregulated in response to auxin, brassinosteroid, cytokinin, and gibberellin treatments in root and shoot tissues. Notably, *OsJaDof10.1* significantly up-regulated in roots and shoots of abscisic acid-treated seedlings. Likewise, *OsJaDof1.1* and *OsJaDof2.1* significantly down-regulated in roots and shoots of jasmonic acid-treated seedlings. Overall, these results indicate the vital roles of Dof genes in phytohormones perception, signal transduction, and downstream effects to stimulate the rice defense response mechanisms against different stresses.

Similarly, the expression patterns of *O. sativa indica Dof*s were investigated in two contrasting rice genotypes for low light responsiveness (**Figure 6B**). Consistent with *O. sativa japonica Dofs* expression patterns under hormone stress, the majority of the *OsInDof*s (>66%) were deregulated after exposure to low light treatments. Interestingly, all these genes were up-regulated in low light tolerant rice (Swarnaprabha), with exception of a few genes which marginally down-regulated. Contrarily, only 44% of all *OsInDof*s were deregulated in low light-sensitive rice (IR8) and a majority of these (63%) were downregulated after exposure to at least one low light treatment (**Figure 6B**). Collectively, these results suggest that accelerated expressions of *OsInDof*s are detrimental for maintaining optimum photosynthetic processes under low light conditions.

Furthermore, *in-silico* expression profiles were validated by investigating qRT-PCR-based relative expression patterns under low light, high-temperature, and low-temperature treatments (**Figure 6C**; **Supplementary Table S10**). After one week of low light treatment (20% of control; 60 µmolm^-2^s^-1^), the majority of the investigated genes were significantly expressed in both leaf and stem tissues. Notably, nearly all genes were strongly expressed in stems, whereas genes of at least four Dof subgroups (I, II, IV, and VII) were also highly expressed in leaves as compared with control. As expected, the majority of these strongly expressed genes also showed higher relative expression profiles in low light tolerant rice (**Figure 6B**). Similarly, nearly 50% of genes were significantly expressed in leaves after exposure to one week of high temperature (40 ± 1°C) treatment during 14h daylight period (**Figure 6C**). Likewise, 11 investigated genes out of 14 were also highly expressed in at least one tested tissue after exposure to one week of low light (15 ± 1°C) treatment. Notably, at least five genes (*OsInDof1.4*, *OsInDof1.5*, *OsInDof2.3*, *OsInDof3.3*, *OsInDof4.2*) showed significantly higher relative expressions in leaves and two genes (*OsInDof3.1*, *OsInDof7.2*) showed significantly higher relative expressions in stems under all three stress treatments (**Figure 6C**). Together, these wet-lab-based expression profiles strongly support the *in-silico* results and suggest multifarious roles of Dof genes in regulating the growth and development and tolerance to abiotic stresses.

### Significant haplotypes for early flowering in rice

**Figure 7:**
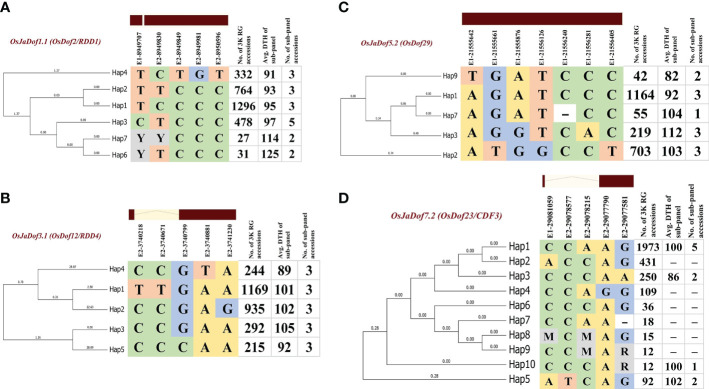
Superior haplotypes of four orthologous genes across genus *Oryza* including **(A)** *OsJaDof1.1*, **(B)** *OsJaDof3.1* **(C)** *OsJaDof5.2*, and **(D)** *OsJaDof7.2* significantly associated with days to heading data from field evaluation. The genomic positions of non-synonymous substitutions in the coding region of genes are indicated above the haplotype blocks with E1 and E2 indicating exon 1 and exon 2, respectively followed by the genomic position of SNP-based polymorphism.

Dof genes have been recently reported to be strongly associated with heading date in rice (Huang et al., 2020). We took advantage of previously reported 3,010 rice genomes variation and phenotypic data (Wang et al., 2020), and mined haplotype blocks significantly associated with days to heading (DTH) trait in coding regions of four orthologous Dof genes harbored by all ten rice genomes (**Figure 7**; **Supplementary Table S11**). Furthermore, we also validated the identified haplotypes with field experiments data across two consecutive growing seasons (**Supplementary Table S12**). In total, we identified 26 haplotypes (six for *OsJaDof1.1*, five for *OsJaDof3.1* and *OsJaDof5.2* each, and ten for *OsJaDof7.2*) with significant variation in their days to heading data. For *OsJaDof1.1*, haplotypes 4 and 2 (Hap4, Hap2) were found to be superior as rice accession harboring these haplotypes have taken 91 and 93 days on average for flowering starting from germination, respectively (**Figure 7A**). Similarly, Hap4 and Hap5 of *OsJaDof3.1* took 89 and 92 days for flowering, respectively (**Figure 7B**). Likewise, Hap9 and Hap1 of *OsJaDof5.2* took 82 and 92 days for flowering, respectively (**Figure 7C**). Whereas Hap2 of *OsJaDof7.2* was found to be superior to all others, however, we could not validate it with field evaluation-based experiments as none of the sub-panel accessions harbored this haplotype, although Hap3 was also found to be superior and validated with field evaluation (**Figure 7D**). Overall, our results indicate that superior haplotypes for early flowering are contributed by wild relatives of rice and will be helpful to broaden the genetic diversity of cultivated rice. Moreover, these alleles can also be used to develop varying photoperiod-specific rice varieties.

### Species evolution and gene flow among ten *Oryza* genomes

**Figure 8:**
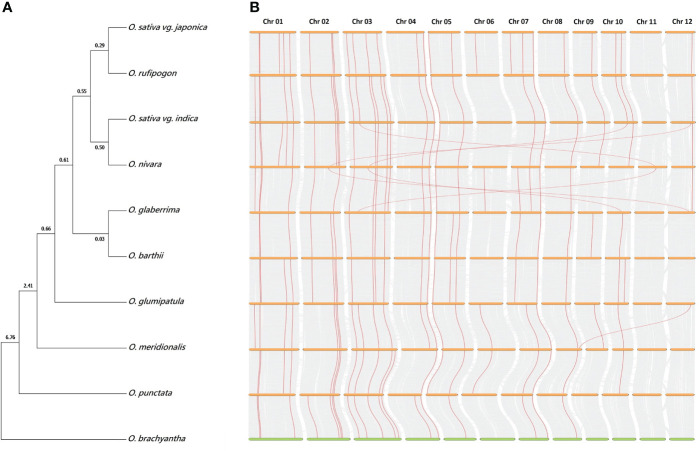
Evolutionary phylogenetic relationships and chromosomal synteny analysis among *Oryza* genomes. **(A)** Species phylogeny was inferred after MAFFT v7 multiple sequence alignment (Katoh et al., 2018) and neighbor-joining real-time tree creation with MEGA7 (Kumar et al., 2016) using coding sequences of five orthologous Dof genes present across all ten *Oryza* genomes. *O. brachyantha* was considered as an outgroup while estimating evolutionary divergence time in million years (above tree nodes). **(B)** Chromosomal synteny analysis among adjacent species was carried out with TBtools (Chen et al., 2020) by using GFF3 and peptide sequence files of ten *Oryza* genomes. Gene links indicate orthologous Dof genes and their chromosomal locations among different genomes.

The exact evolutionary phylogenetic relationships among *Oryzae* species remained elusive, particularly due to phylogeny discordance among published reports (Degnan and Rosenberg, 2009; Zwickl et al., 2014). In this study, we inferred a single-species phylogeny using five single-copy orthologs from all ten rice genomes (**Figure 8A**). We noted two independent origins of Asian cultivated *japonica* and *indica* rice, whereas African cultivated *O. glaberrima* rice shared a closer evolutionary phylogenetic relationship with South American *O. glumipatula* rice, rather than the *O. sativa* complex. The real-time method-based time tree indicated a more recent divergence of two African rice genomes (~0.03 Mya), followed by *O. sativa* vg. *japonica* divergence from *O. rufipogon* (~0.29 Mya) and *O. sativa* vg. *indica* divergence from *O. nivara* (~0.50 Mya). As expected, the eight AA genomes (*O. sativa indica* and *japonica*, *O. nivara*, *O. rufipogon*, *O. glaberrima*, *O. barthii*, *O glumipatula*, and *O. meridionalis*) were found to be recently diverged from the BB genome (*O. punctata*), as compared with FF genome (*O. brachyantha*).

Furthermore, we also investigated genetic conservation and synteny among *Oryza* genomes. Notably, dual synteny plots highlighted >90% synteny among all ten genomes (**Figure 8B**), as nearly all ancestral wild species Dof genes had orthologs in descendant cultivated species. We noted a higher turnover of Dof genes among AA genomes, particularly among cultivated species complex, and few translocations between *O. sativa* vg. *indica* and *O. nivara* and between *O. nivara* and *O. glaberrima* Dof genes. Collectively, these results reveal higher genetic conservation across the *Oryza* genomes.

## Discussion

### Recent progress towards identification and characterization of Dof genes in rice

Dof genes belong to plant kingdom-specific transcription factor family that play important roles during the growth and development of nearly all plant species. In the recent past, significant progress was made towards comprehensive genome-wide identification and functional characterization of Dof family genes in rice which could facilitate marker-assisted development of rice cultivars. For example, two more recent genome-wide studies independently identified a total of 30 *Dof*s in the *O. sativa japonica* genome and reported their phylogenetic relationships, gene structures, conserved motifs, gene duplications, and expression patterns in different tissue under normal and cold stress conditions (Khan et al., 2021; Liu et al., 2021). Our results demonstrated the presence of copy number variation of Dof genes on some chromosomes of among *indica* rice genomes. The copy number variation of Dof genes in wheat has been shown to increase the stem solidness (Nilsen et al., 2020) which can ultimately be exploited for the development of wheat stem sawfly resistance.

Rice *OsDof12/RDD4* has been shown to be a genetic regulator of plant architecture, as its overexpression could lead to shortened plant height, erect leaves, narrow leaf blades, and smaller panicles (Wu et al., 2015). *RDD1* and *RDD4* promote efficient uptake and accumulation of several nutrients such as NH_4_^+^, Na^+^, SO_4_^2‾^, Cl^‾^, PO_4_^3^‾, and sucrose under low-nutrient conditions and contribute to increased grain productivity (Iwamoto and Tagiri, 2016; Iwamoto et al., 2021). *OsDof4* regulates photoperiodic flowering under short and long days (Wu et al., 2017b). *OsDOF18* affects nitrogen use efficiency in roots by inducing ammonium transporters and mediating the ammonium transport and nitrogen distribution (Wu et al., 2017a). *OsDOF11* modulates sugar distribution in different plant organs during the pathogenic invasion by accelerating the expression of *SUT* and *SWEET* genes (Wu et al., 2018). *OsDOF24* delays leaf senescence by deactivating jasmonate biosynthetic pathways (Shim et al., 2019). *OsDOF15* positively regulates primary root elongation under salt stress by restricting ethylene biosynthesis in developing roots (Qin et al., 2019). Moreover, minor effects of 11 *Dof*s contribute diverse functions in promoting and suppressing heading dates under contrasting photoperiodic conditions (Huang et al., 2020). These recent advancements, as well as the results presented in this study, could contribute significantly toward the genomics-assisted development of tailor-made rice cultivars.

### Dof genes galore among *Oryza* genomes

Wild crop relatives constitute an important genetic reservoir for improvement due to their tolerance to different stresses and wider adaptability (Jena, 2010; Atwell et al., 2014). Harnessing superior traits for crop improvement hold enormous promises, as revealed by the introgression of bacterial resistance from wild rice to cultivated rice (Song et al., 1995). To precisely harness the genetic potential of wild relatives, a clear understanding of genomic conservation and diversification is crucial. Taking advantage of recently sequenced wild relatives of cultivated rice (Stein et al., 2018), we comprehensively identified 238 full-length protein-encoding *Dof* genes through genome-wide scans in ten *Oryza* genomes (**Figures 2A**, **D**; **Supplementary Table S3**). Previously, Dof genes were only identified in three cultivated (*O. sativa indica* and *japonica*, *O. glaberrima*) and one wild relative of rice (*O. brachyantha*) (Lijavetzky et al., 2003; Jacquemin et al., 2014; Khan et al., 2021; Liu et al., 2021), ignoring other important wild rice species. Furthermore, variable number of Dof phylogenetic subgroups were reported in those studies, thus lacking consistency in phylogenetic classification. Based on evolutionary phylogenetic relationships, *Arabidopsis* Dof genes were classified into seven subgroups (I–VII) (Yanagisawa, 2002), which serve as a reference for Dof phylogenetic classification in later studies. The *Oryza* Dof genes identified in this study were also clustered into seven distinct subgroups showing distant orthologous relationships with *Arabidopsis* genes in each subgroup (**Figure 2A**). Interestingly, two subgroups (II and V) were overrepresented in all investigated genomes, with *O. brachyantha* harboring the least gene count and entirely lacking subgroup V, as well as subgroup III members (**Figure 2D**). The reason could be the shortest genome size and extended evolutionary history, as *O. brachyantha* diverged >15 million years ago, whereas all other *Oryza* genomes diverged ≤ 6.76 million years ago (Stein et al., 2018). Conversely to previous reports on genome-wide identification of Dof genes in *O. sativa japonica* (Lijavetzky et al., 2003; Khan et al., 2021; Liu et al., 2021), only 25 non-redundant *OsJaDofs* containing complete Dof domain were identified in this study (**Figures 2A**, **D**; **Supplementary Table S3**), highlighting those previous studies also included incomplete Dof domain-containing genes. Overall, our results demonstrate an abundance of wider genetic and genomic resources in domesticated and wild rice relatives for further crop improvement.

### Structural and functional diversity among Dof subgroups

Dof genes were reported to be occurred in ancient days, even before the diversification of angiosperms (Yanagisawa, 2002). Because of an extended evolutionary history, different Dof subgroups might come to play diversified roles. This could be largely due to amino acid substitutions in genomic regions outside the conserved Dof domain. Therefore, it is not unexpected that different Dof subgroups show distinct and/or overlapping functions (Yanagisawa, 2004). In this study, we also observed wider genetic diversity in iso-electric points and molecular weights of different Dof subgroups (**Figures 2B**, **C**), which was supported by conserved motifs and gene structure analyses (**Figure 3** and **Supplementary Figures S1A–S1H**). Members of different subgroups demonstrated a prevalence of structural diversification between them, however, conservation among members of the same subgroup. Moreover, subgroup-specific gene duplications among Dofs of ten Oryza genomes (**Figure 4**) and gene expression profiles in response to phytohormones and low light treatments (**Figures 6A**, **B**), strongly reinforced the current results. Thus, the current catalog of Oryza Dof genes might disclose both the subgroup-specific and species-specific structural and functional diversity that is expediating with the passage of the evolutionary period.

### *Dof*s as transcriptional regulators in plant growth and development and hostile environments

Dofs are plant-specific transcription factors that bind to the specific CAREs present in their promoter regions and regulate diverse biological, physiological, and molecular functions both at transcriptional and post-transcriptional levels (Yanagisawa, 2004). Previous reports have shed light on their multifarious roles in regulating the growth and development, as well as resistance or tolerance to biotic and abiotic stresses throughout the plant kingdom as briefly reviewed in previous reports (Gupta et al., 2015; Waqas et al., 2020; Zhuo et al., 2020). In this study, a variety of CAREs corresponding to diverse potential functions, especially tolerance to abiotic and hormone stresses, were identified in promoters of *Dof* genes of all *Oryza* species (**Supplementary Tables S5**, **S6** **)**. Furthermore, the majority of the genes of the two most important cultivated *Oryza* species (*O. sativa indica* and *japonica*) were significantly deregulated in response to low light and phytohormone treatments (**Figures 6A**, **B**), strongly backing the promoter analysis results. Recent reports have also revealed their regulatory roles in cold, heat, and shade tolerance (Sekhar et al., 2019; He et al., 2020; Liu et al., 2021) confirming their diverse roles in rice growth and development and wider adaptability in hostile environments. Collectively, these results strongly support the notion about *Dof*s act as dominant transcriptional regulators of plant life cycle and wider adaptability. However, further deeper insights at gene regulatory networks and systems biology levels are required to confirm this hypothesis.

### Superior haplotypes for haplotype-based earliness breeding in rice

The genetic variation and phenotypic agronomic data of the 3K RG panel is a treasure trove for molecular rice breeders for exploiting haplotype diversity (Sun et al., 2017; Wang et al., 2020). In this study, we identified significant haplotypes associated with days to heading field data across the 3K RG panel and validated these haplotypes with a two-year field evaluation of a representative sub-panel (**Figure 7**; **Supplementary Tables S9, S10**). The superior haplotypes predominately originated from wild rice accessions and flowered significantly earlier than all other identified haplotypes. These findings are backed by the significant contribution of Dof genes to the missing heritability of heading dates in rice (Huang et al., 2020). Introgression of these superior haplotypes through modern haplotype-led breeding could assist in designing early maturing rice cultivars under fluctuating climatic conditions. However, multi-location field data-based confirmatory studies involving a larger number of 3K RG representative accessions need to be carried out before successfully harnessing the potential of these superior haplotypes in applied research programs. Systems-level deeper insights into the economically important traits, underlying genes, and associated epigenetic factors could greatly facilitate the development of customizable rice ideotypes. Once realized, this strategy would accelerate the way toward designer rice plants.

### Rapid species diversification and higher gene flow facilitate wider adaptability and further crop improvement

Species diversity among important crop plants is of paramount importance for their broader cultivation followed by adaptation and holds promise for further crop improvement. Here, we resolved controversial areas of *Oryza* species phylogeny by untangling the complex evolutionary phylogenetic relationships among closely related species (Degnan and Rosenberg, 2009; Zwickl et al., 2014) (**Figure 8A**). Our results highlighted rapid species diversification rate of approximately 0.50 million years per new species among AA genomes, which is at par with many other rapidly evolving taxa (Madriñán et al., 2013; Zhu et al., 2014). For example, we found recent independent origins of two widely cultivated subspecies *indica* and *japonica*, whereas another cultivated species *O. glaberrima* revealed more recent divergence from wild *O. barthii* rice and higher evolutionary relatedness with South American wild rice *O. glumipatula* (Stein et al., 2018). However, despite rapid species diversification, footprints of higher gene flow are still detectable, as nearly all wild Dof genes had orthologs in recently evolved cultivated species (**Figure 8B**). This rapid diversification coupled with higher genetic conservation and gene flow among wild and cultivated species facilitates wider adaptability under changing climatic conditions and provides special opportunities for broader cultivation and further crop improvement.

## Conclusion

In this study, we reported a complete catalog of full-length Dof family genes in ten cultivated and wild *Oryza* genomes through genome-wide scanning. A total of 238 Dof genes were classified into seven distinct phylogenetic subgroups sharing structural and functional diversity. Our results revealed exclusive localization of duplicated gene pairs on 2^nd^, 3^rd,^ and 7^th^ chromosomes and Dof domain sequences potentially containing target sites for miR2927 across the *Oryza* genomes. Transcriptional profiles in different tissues under phytohormones and low light treatments strongly support that Dof genes act as dominant transcriptional regulators during rice growth and development and wider adaptation under changing environments. Furthermore, superior haplotypes significantly associated with early flowering across the 3K RG panel were reported and validated with two years of field experiments based on the morphometric evaluation that could be exploited through haplotype-led breeding for fast-track development of early flowering rice cultivars. Finally, we resolved *Oryza* species’ phylogeny discordance and observed higher genetic conservation and gene flow among *Oryzae* species which provide better opportunities for wider adaptability and further crop improvement.

## Data availability statement

The datasets presented in this study can be found in online repositories. The names of the repository/repositories and accession number(s) can be found in the article/**Supplementary Material**.

## Author contributions

RA and JL conceived the idea. JT, QR, AR, JL and RA designed the experiments. JT, QR, AR, SA and MR retrieved and curated the data. JT, QR, AR, SA, MR and FK performed analyses. JT, QR and AR performed formal analyses and drafted the manuscript. MJ, ZA, IK, JL and RA reviewed and edited the manuscript. IK, JL and RA provided resources, supervised the experiments, and acquired funding for research. All authors have read and approved the final manuscript.

## Funding

This work was supported by Agricultural Sciences and Technologies Innovation Program of the Chinese Academy of Agricultural Sciences (CAAS); and Higher Education Commission of Pakistan (HEC) through their grant of Precision Agriculture and Analytics Lab (PAAL) under National Centre in Big Data and Cloud Computing (NCBC).

## Acknowledgments

The authors are thankful to the Rice Research Institute, Kala Shah Kaku, Sheikhupura, Pakistan for providing institutional support for carrying out field experimentation. The infrastructural support from Centre for Advanced Studies in Agriculture and Food Security (CAS-AFS), at University of Agriculture Faisalabad, Pakistan is also grateful acknowledged.

## Conflict of interest

The authors declare that the research was conducted in the absence of any commercial or financial relationships that could be construed as a potential conflict of interest.

## Publisher’s note

All claims expressed in this article are solely those of the authors and do not necessarily represent those of their affiliated organizations, or those of the publisher, the editors and the reviewers. Any product that may be evaluated in this article, or claim that may be made by its manufacturer, is not guaranteed or endorsed by the publisher.
