# Supplementary material for: Exploration of the genomic atlas of Dof transcription factor family across genus Oryza provides novel insights on rice breeding in changing climate
Source: Front Plant Sci. 2022 Nov 3;13:1004359. doi: 10.3389/fpls.2022.1004359 (PMC9671800; doi:10.3389/fpls.2022.1004359)
Supplement: Supplementary file 2 [file DataSheet_2.docx]

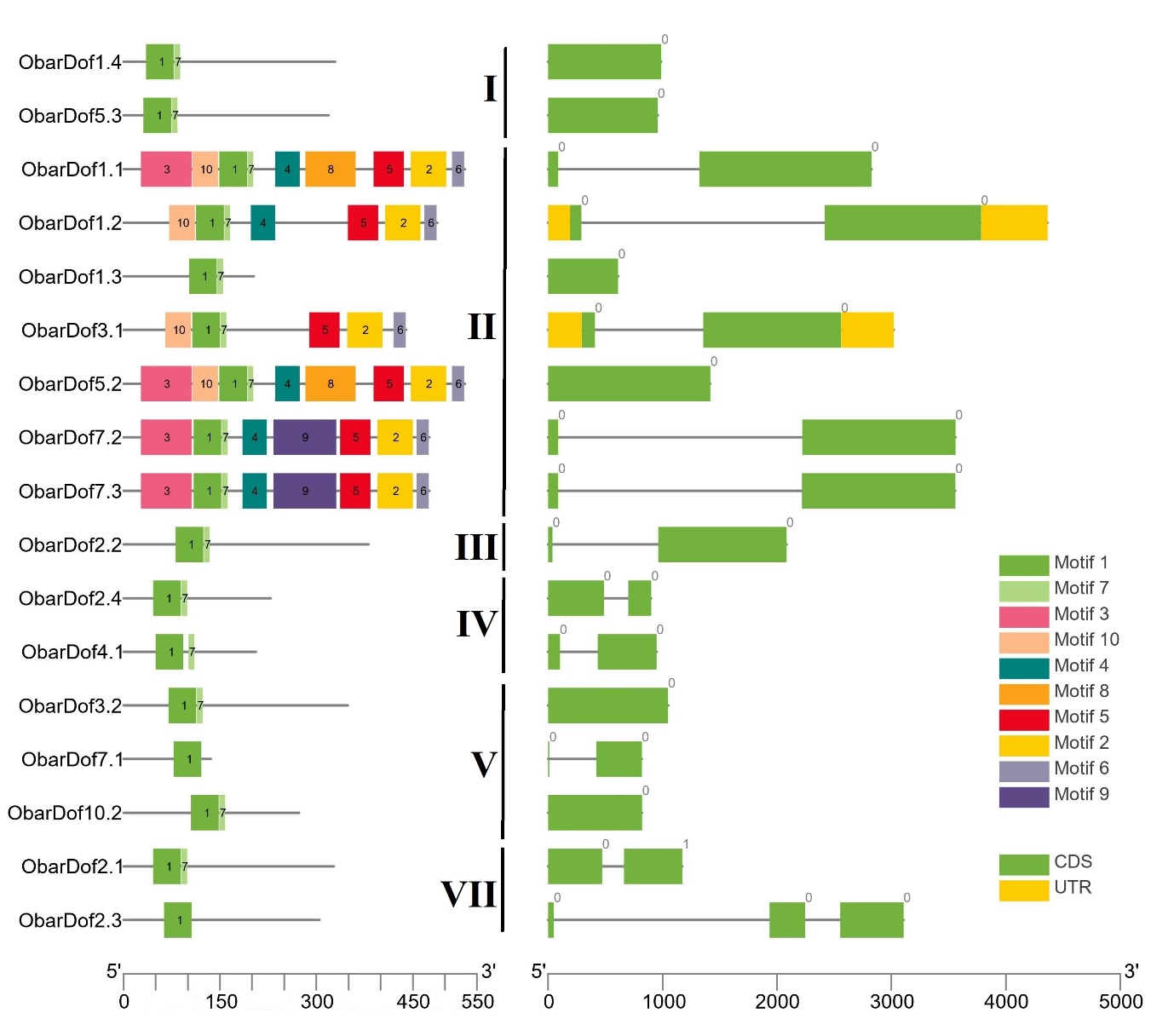


**Figure S1A:** Conserved motifs (left panel) and gene structure analysis (right panel) of *O. bartti* Dof genes. Different Dof subgroups are indicated with roman numbers in center.


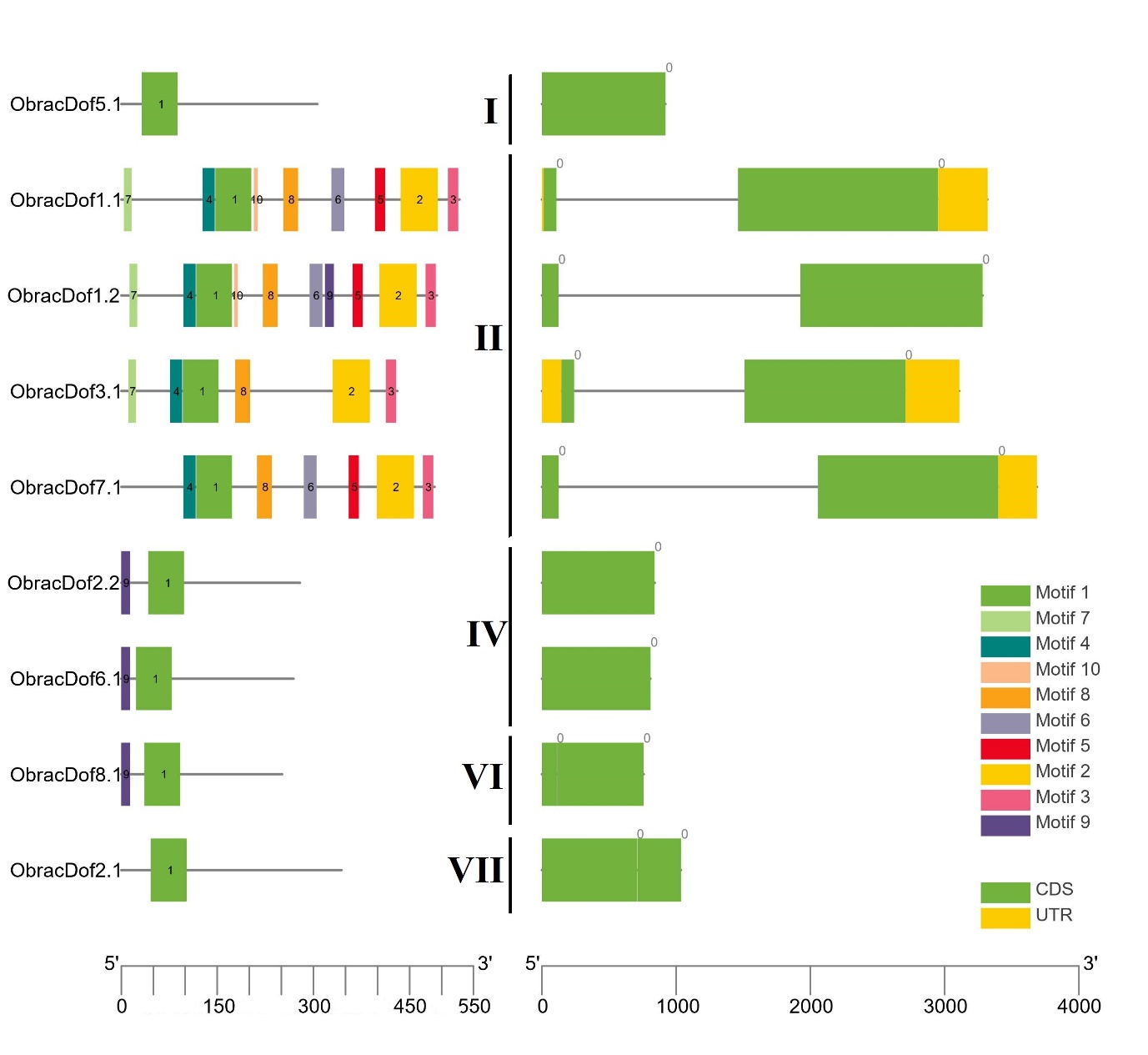


**Figure S1B:** Conserved motifs (left panel) and gene structure analysis (right panel) of *O. brachyantha* Dof genes. Different Dof subgroups are indicated with roman numbers in center.


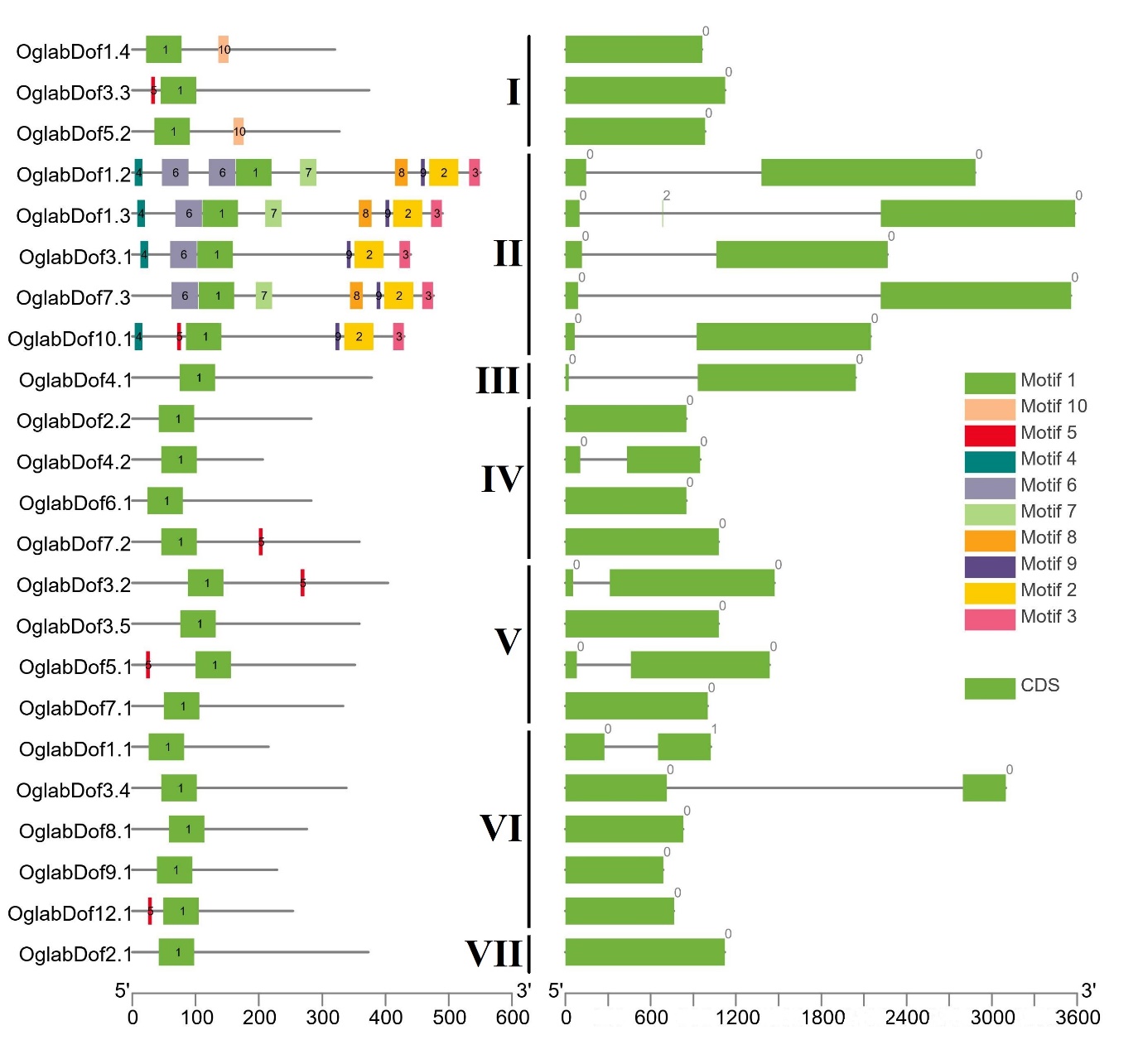


**Figure S1C:** Conserved motifs (left panel) and gene structure analysis (right panel) of *O. glaberrima* Dof genes. Different Dof subgroups are indicated with roman numbers in center.

**Figure S1D:** Conserved motifs (left panel) and gene structure analysis (right panel) of *O. glumipatula* Dof genes. Different Dof subgroups are indicated with roman numbers in center.


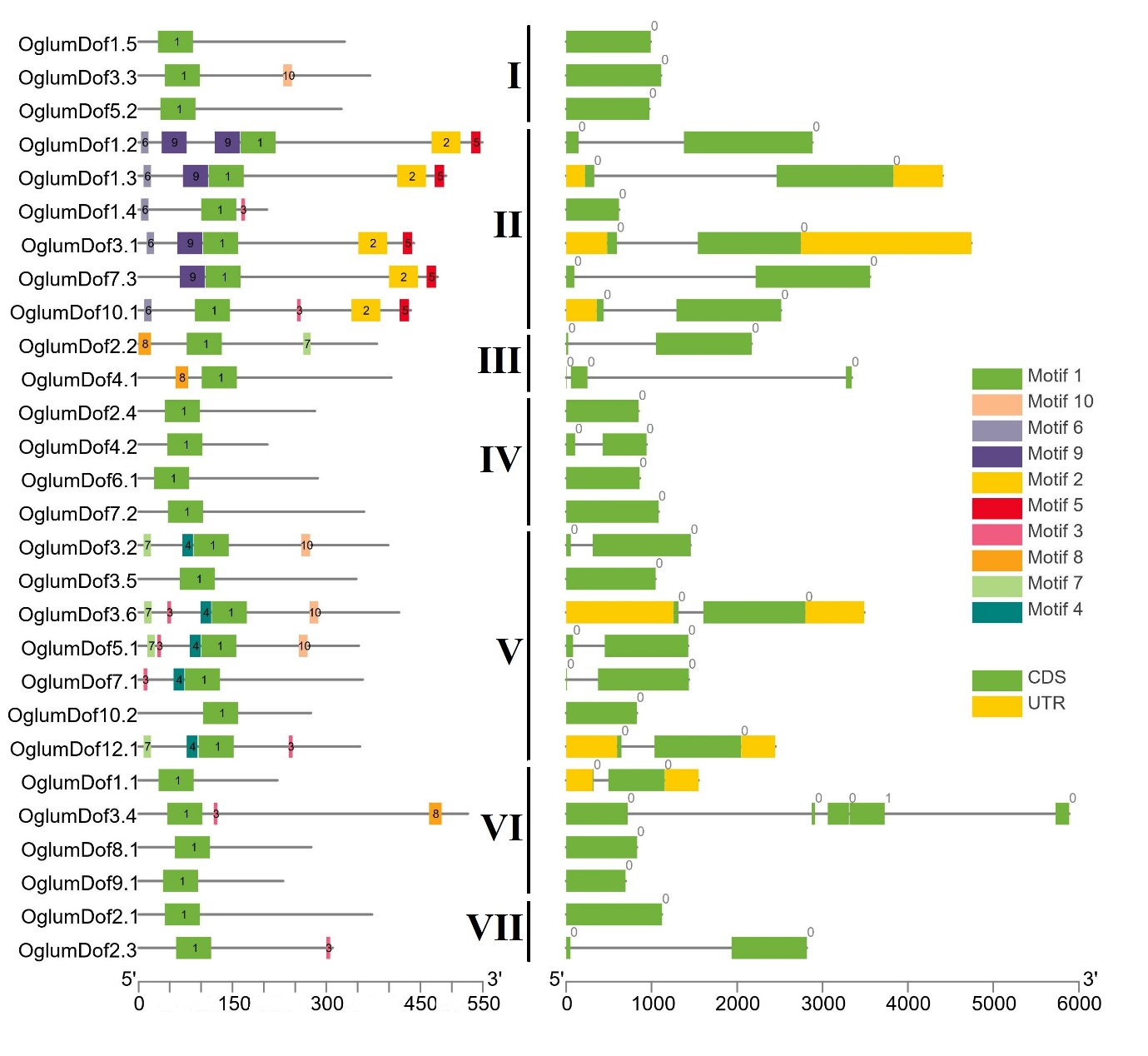


**Figure S1E:** Conserved motifs (left panel) and gene structure analysis (right panel) of *O. meridionalis* Dof genes. Different Dof subgroups are indicated with roman numbers in center.


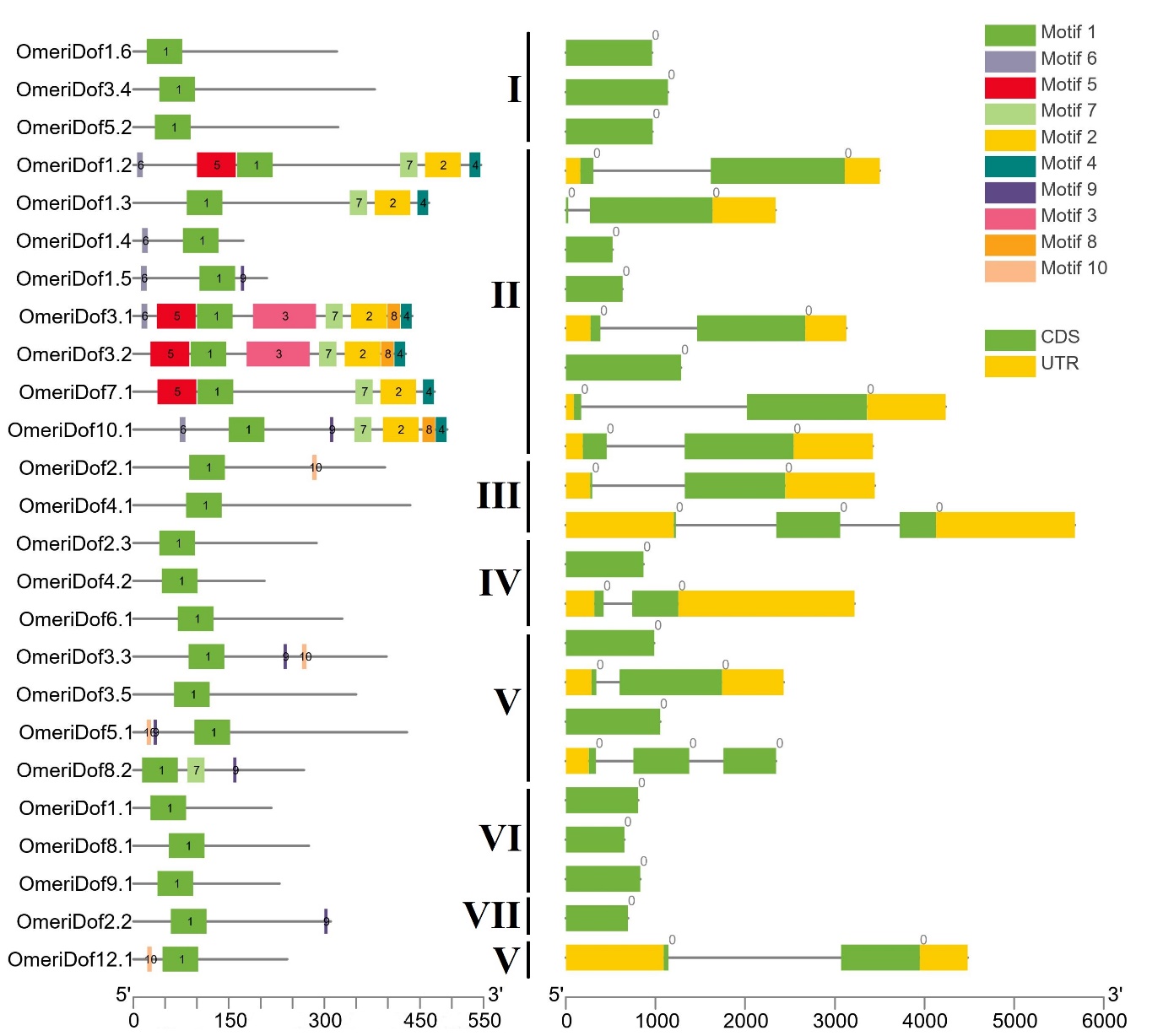


**Figure S1F:** Conserved motifs (left panel) and gene structure analysis (right panel) of *O. nivara* Dof genes. Different Dof subgroups are indicated with roman numbers in center.


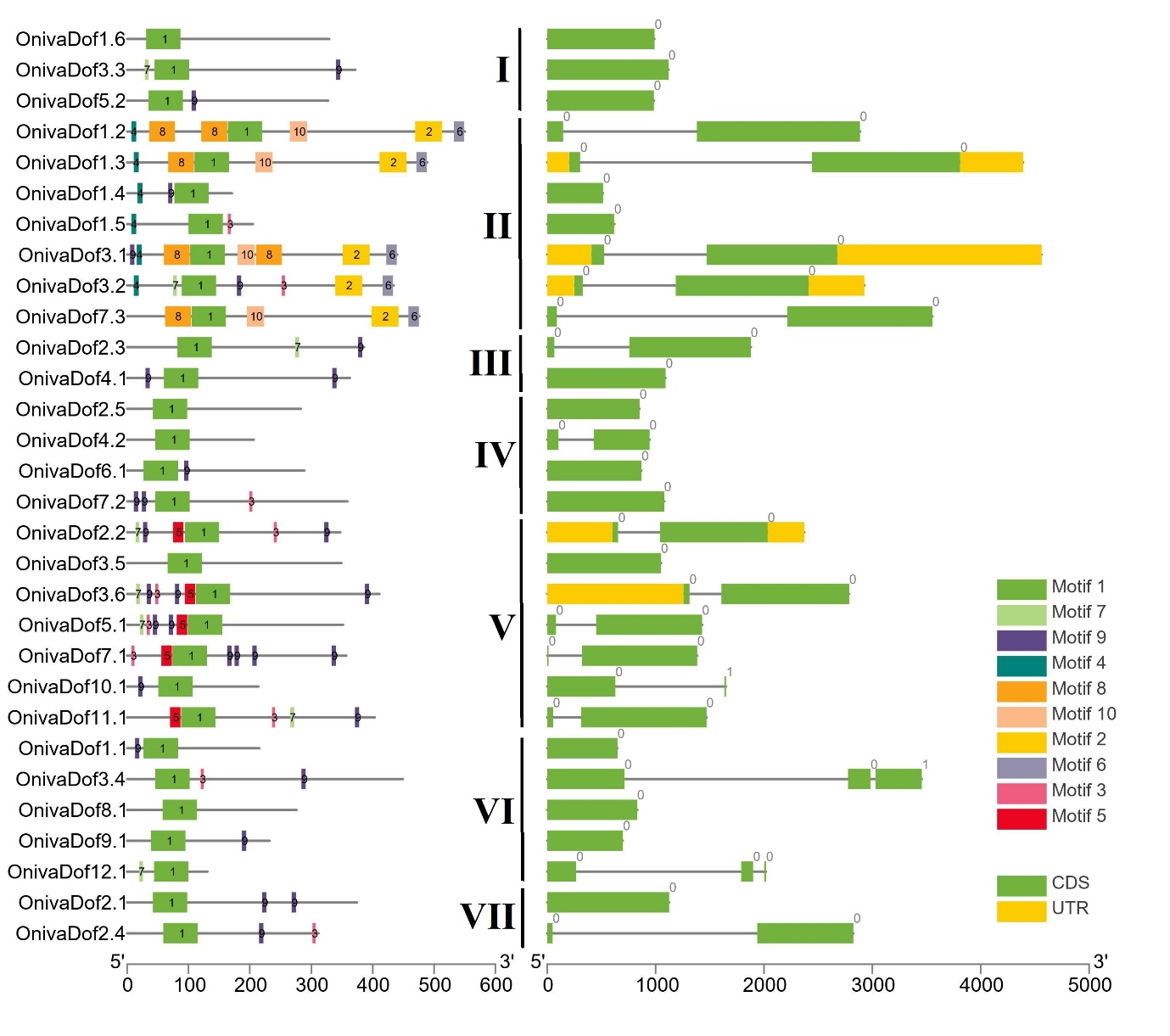


**Figure S1G:** Conserved motifs (left panel) and gene structure analysis (right panel) of *O. punctata* Dof genes. Different Dof subgroups are indicated with roman numbers in center.


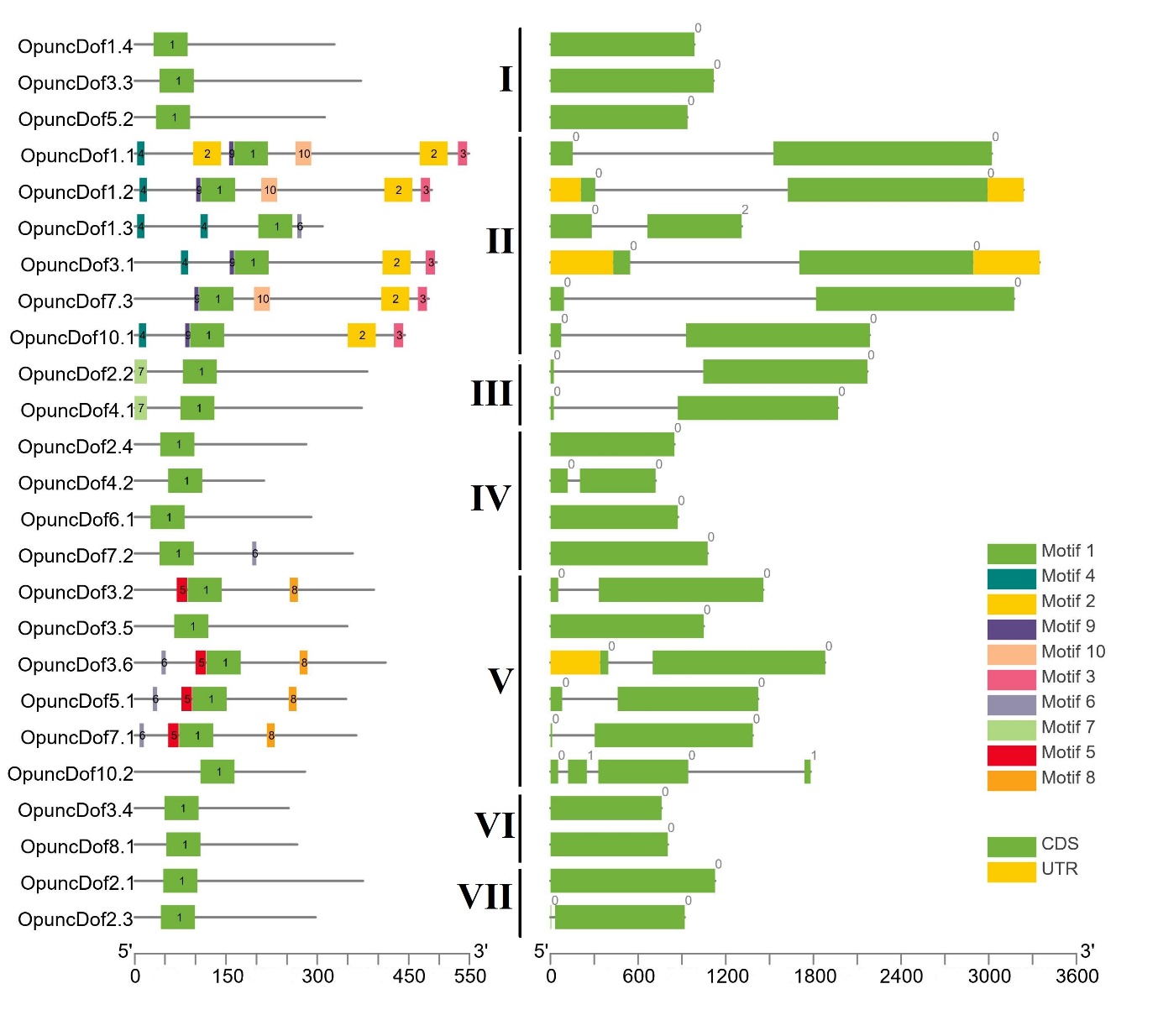


**Figure S1H:** Conserved motifs (left panel) and gene structure analysis (right panel) of *O. rufipogon* Dof genes. Different Dof subgroups are indicated with roman numbers in center.


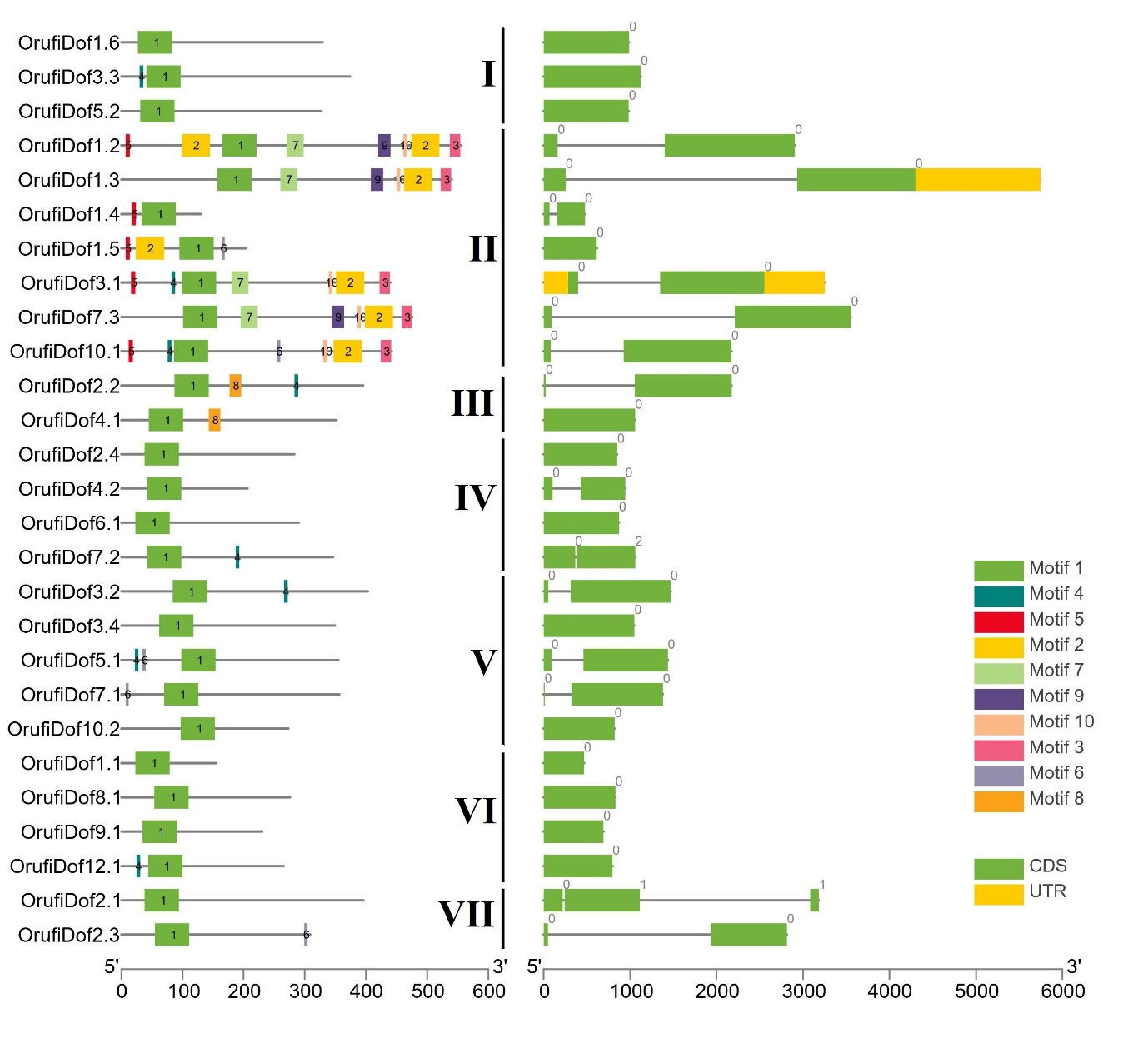


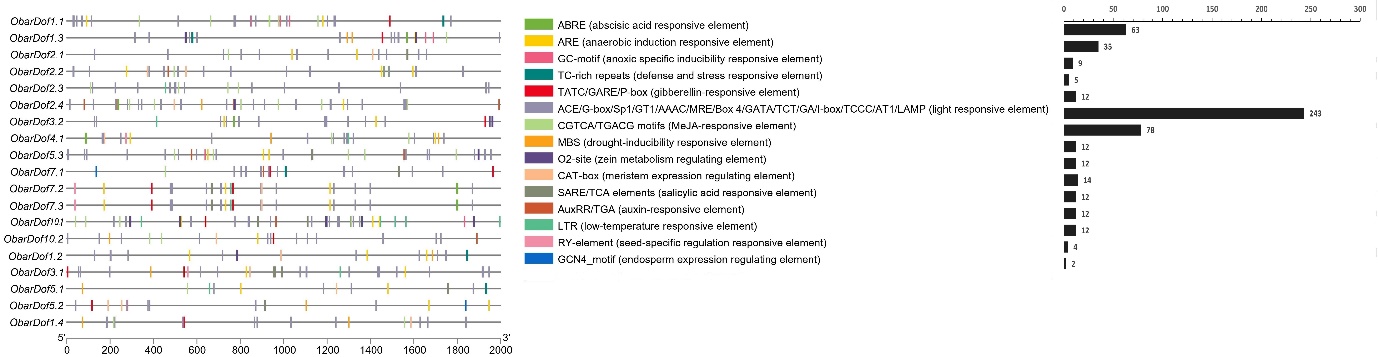


**Figure S2A:** Distribution and frequency of cis-acting regulatory elements in promoter regions of *O. bartti* Dof genes.


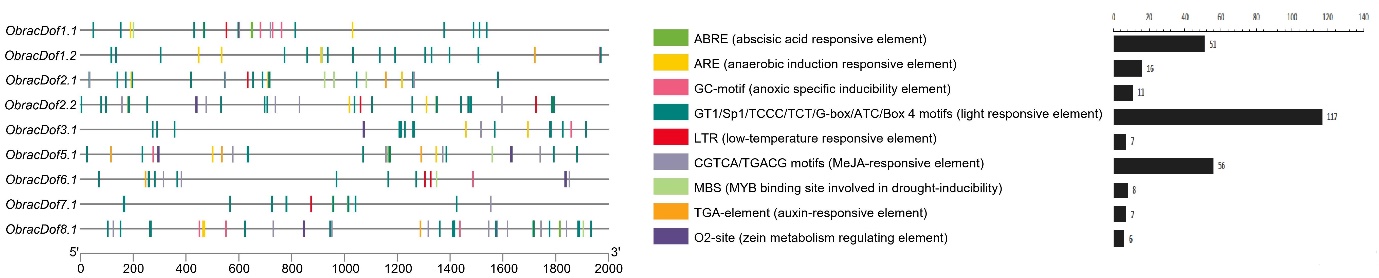


**Figure S2B:** Distribution and frequency of cis-acting regulatory elements in promoter regions of *O. brachyantha* Dof genes.


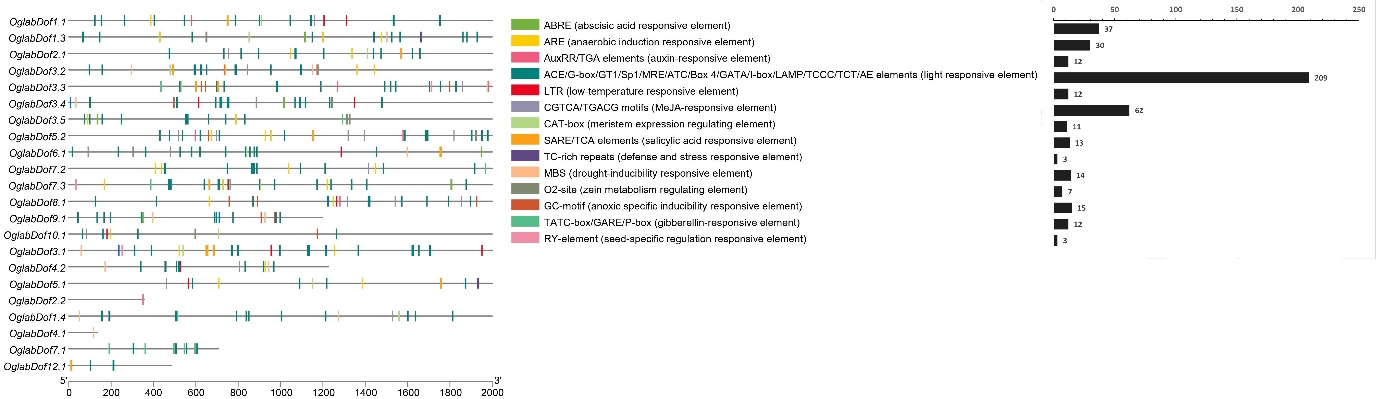


**Figure S2C:** Distribution and frequency of cis-acting regulatory elements in promoter regions of *O. glaberrima* Dof genes.


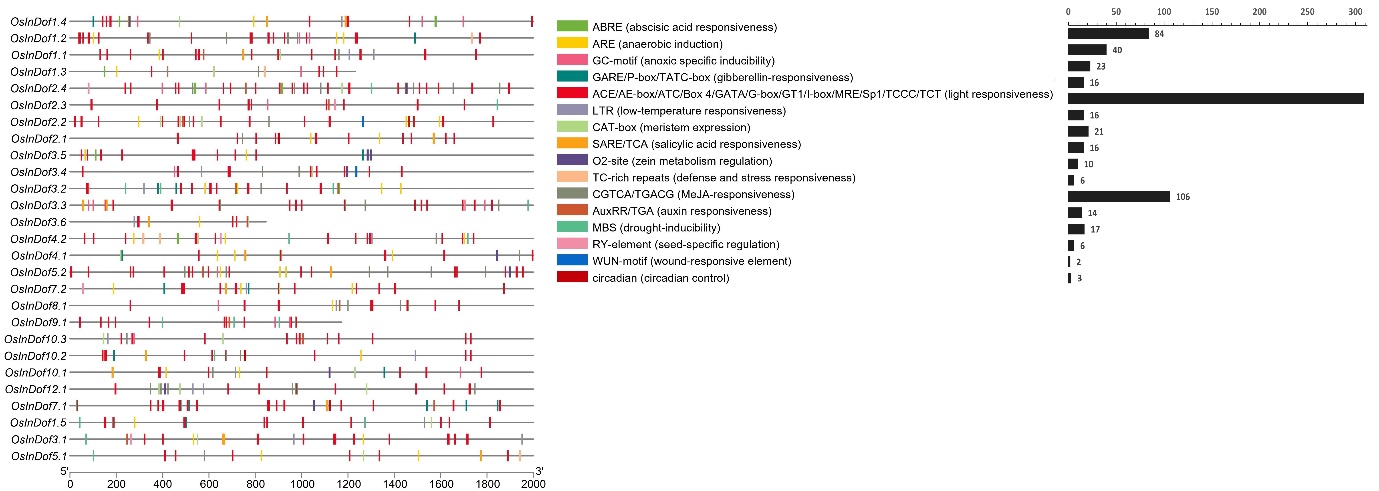
**Figure S2D:** Distribution and frequency of cis-acting regulatory elements in promoter regions of *O. glumipatula* Dof genes.
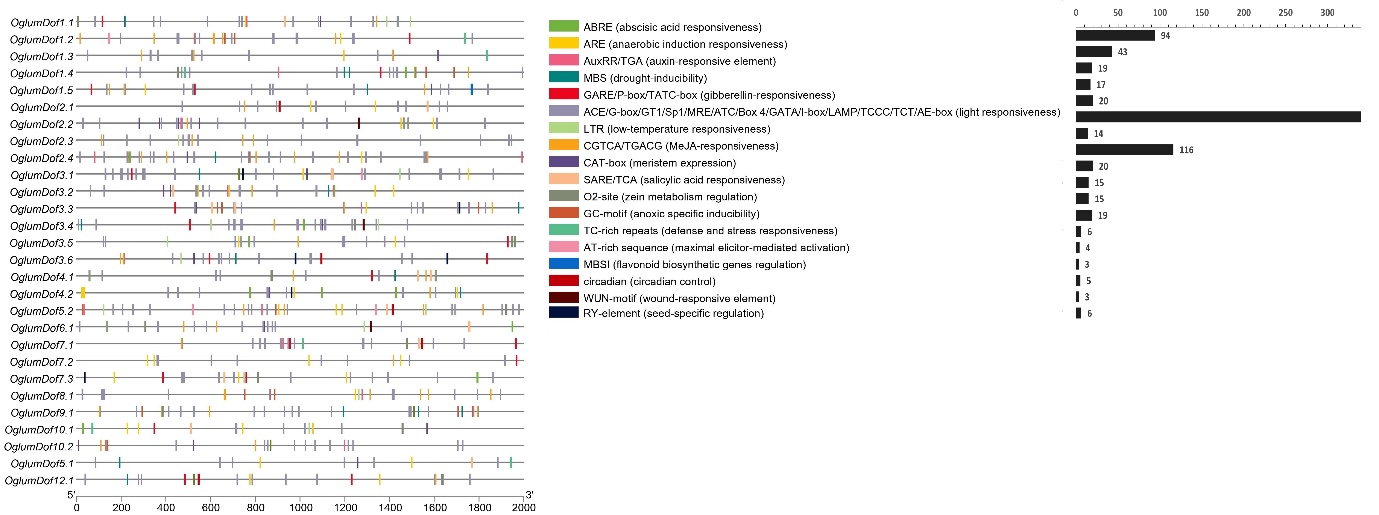


**Figure S2E:** Distribution and frequency of cis-acting regulatory elements in promoter regions of *O. sativa vg. indcia* Dof genes.


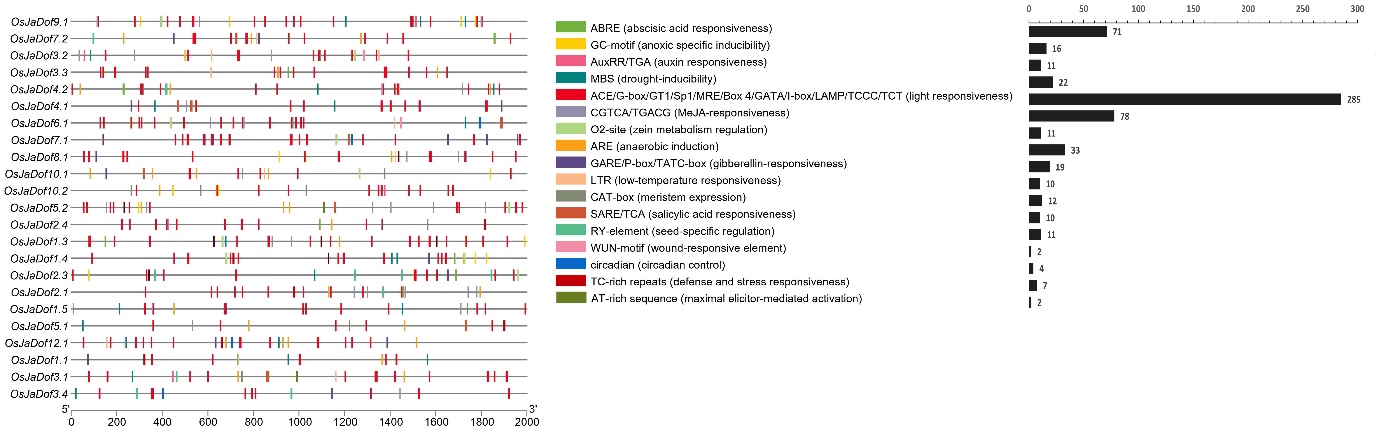


**Figure S2F:** Distribution and frequency of cis-acting regulatory elements in promoter regions of *O. sativa vg. japonica* Dof genes.


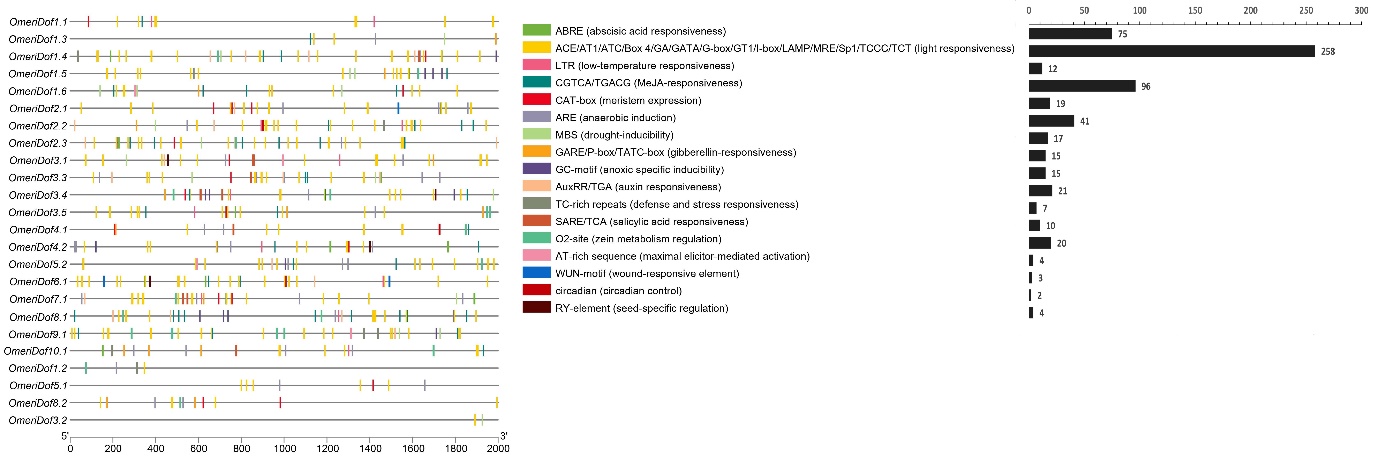


**Figure S2G:** Distribution and frequency of cis-acting regulatory elements in promoter regions of *O. meridionalis* Dof genes.


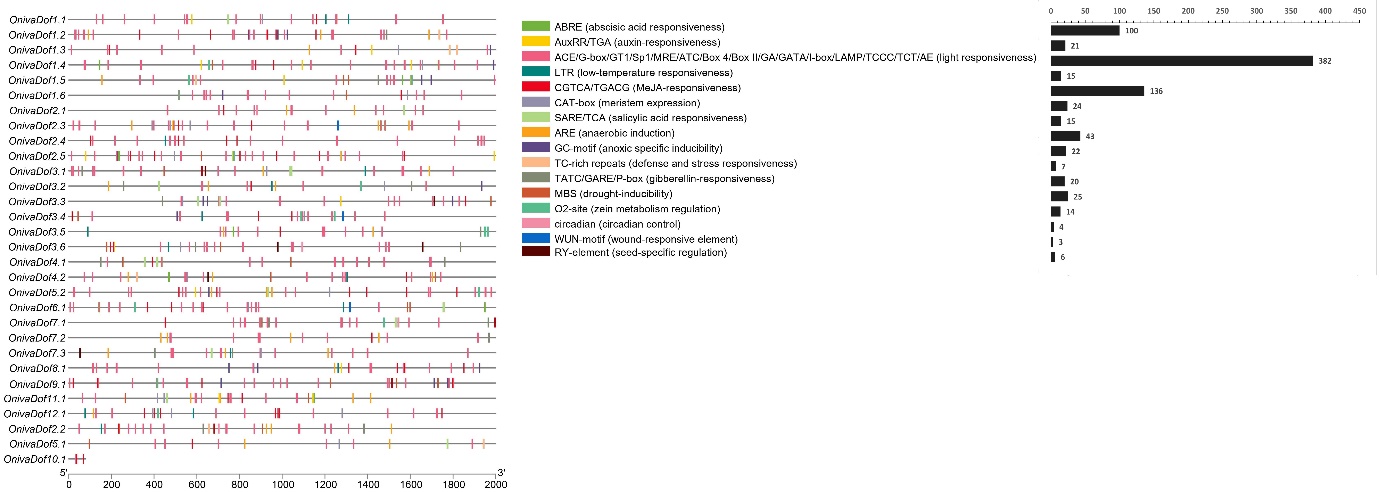


**Figure S2H:** Distribution and frequency of cis-acting regulatory elements in promoter regions of *O. nivara* Dof genes.


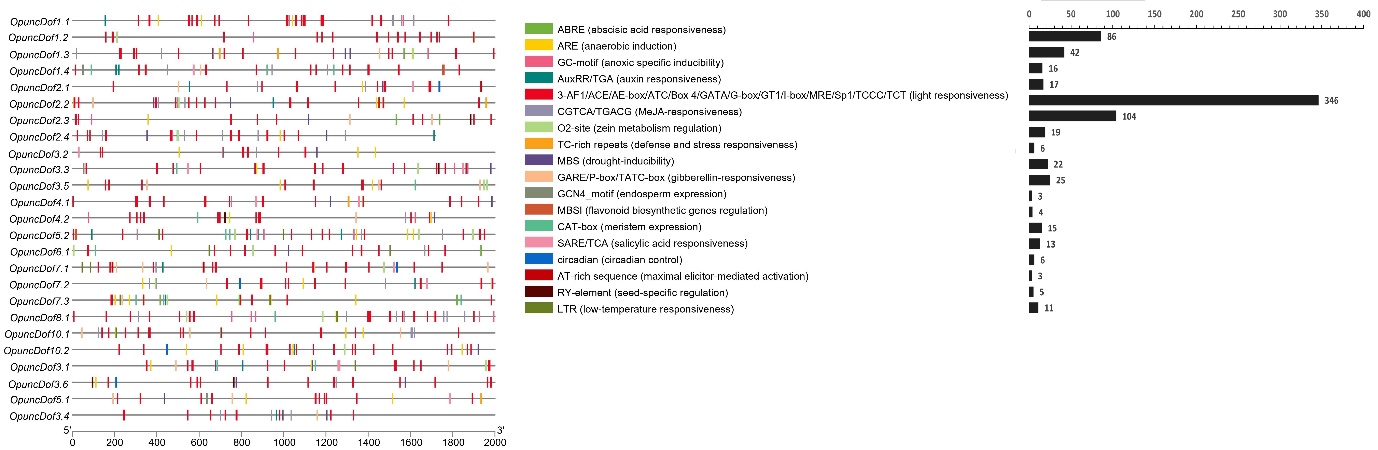


**Figure S2I:** Distribution and frequency of cis-acting regulatory elements in promoter regions of *O. punctata* Dof genes.


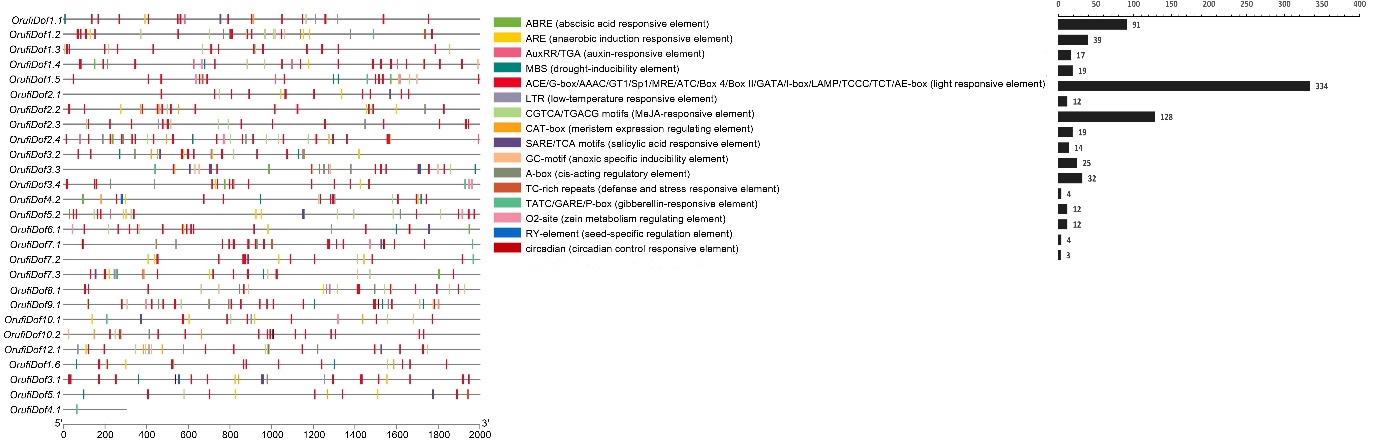


**Figure S2J:** Distribution and frequency of cis-acting regulatory elements in promoter regions of *O. rufipogon* Dof genes.
